# Cardiovascular Effects of Unilateral Nephrectomy in Living Kidney Donors at 5 Years

**DOI:** 10.1161/HYPERTENSIONAHA.120.15398

**Published:** 2021-02-08

**Authors:** Anna M. Price, William E. Moody, Victoria M. Stoll, Ravi Vijapurapu, Manvir K. Hayer, Luca Biasiolli, Chris J. Weston, Rachel Webster, Roman Wesolowski, Kirsty C. McGee, Boyang Liu, Shanat Baig, Luke C. Pickup, Ashwin Radhakrishnan, Jonathan P. Law, Nicola C. Edwards, Richard P. Steeds, Charles J. Ferro, Jonathan N. Townend

**Affiliations:** 1 From the Institute of Cardiovascular Sciences (A.M.P., V.M.S., R.V., M.K.H., B.L., S.B., L.C.P., A.R., J.P.L., R.P.S., N.C.E., C.J.F., J.N.T.), College of Medical and Dental Sciences, University of Birmingham, United Kingdom; 2 Institute of Immunology and Immunotherapy (C.J.W.), College of Medical and Dental Sciences, University of Birmingham, United Kingdom; 3 Institute of Inflammation and Ageing (K.C.M.), College of Medical and Dental Sciences, University of Birmingham, United Kingdom; 4 Department of Nephrology (A.M.P., M.K.H., J.P.L., C.J.F.), University Hospitals NHS Foundation Trust, Birmingham, United Kingdom; 5 Department of Cardiology (W.E.M., V.M.S., R.V., B.L., S.B., L.C.P., A.R., R.P.S., J.N.T.), University Hospitals NHS Foundation Trust, Birmingham, United Kingdom; 6 Department of Biochemistry (R. Webster), University Hospitals NHS Foundation Trust, Birmingham, United Kingdom; 7 Medical Physics (R. Wesolowski), University Hospitals NHS Foundation Trust, Birmingham, United Kingdom; 8 Oxford Centre for Clinical Magnetic Resonance Research, Radcliffe Department of Medicine, University of Oxford, Oxford, United Kingdom (L.B.); 9 Green Lane Cardiovascular Service, Auckland City Hospital, Auckland, New Zealand (N.C.E.).

**Keywords:** blood pressure, kidney, living donors, renal insufficiency, chronic, vascular stiffness

## Abstract

Kidney donation reduces renal function by ≈30% allowing study of the cardiovascular effects of a reduced estimated glomerular filtration rate without comorbidities. We report 5-year results of a longitudinal, parallel-group, blinded end-point study of living kidney donors (n=50) and healthy controls (n=45). The primary end point, left ventricular mass, was measured using cardiac magnetic resonance. Secondary end points, 24-hour ambulatory blood pressure, and pulse wave velocity were measured using validated blood pressure monitors and the SphygmoCor device. Effect sizes were calculated as differences between change from baseline in the donor and control groups. In donors, estimated glomerular filtration rate was 95±15 mL/min per 1.73 m2 at baseline (predonation) and 67±14 mL/min per 1.73 m2 at 5 years. In controls, there was a −1±2 mL/min per 1.73 m2 decline per annum. Change in left ventricular mass at 5 years was not significantly different between donors and controls (mean difference, +0.40 g [95% CI, −4.68 to 5.49] *P*=0.876), despite an initial increase in mass in donors compared with controls at 12 months. Pulse wave velocity, which increased in donors at 12 months, returned to levels not different from controls at 5 years (mean difference, −0.24 m/s [95% CI, −0.69 to 0.21]). Change in ambulatory systolic blood pressure was not different in donors compared with controls (mean difference, +1.91 mm Hg [95% CI, −2.72 to 6.54]). We found no evidence that the reduction in estimated glomerular filtration rate after kidney donation was associated with a change in left ventricular mass detectable by magnetic resonance imaging at 5 years.

## Abstract 2

Supplemental Digital Content is available in the text.

Chronic kidney disease (CKD) is an independent risk factor for cardiovascular morbidity and mortality.^1^ There is an inverse association between estimated glomerular filtration rate (eGFR) and cardiovascular risk.^1^ This risk remains elevated even after adjusting for comorbidities such as hypertension and diabetes.^1,2^ While the threshold eGFR at which cardiovascular risk rises is debatable, many studies have found that risk increases significantly around 60 mL/min per 1.73 m^2^.^3,4^ Although traditional atherosclerotic risk factors commonly accompany CKD, coronary events account for little of the excess mortality.^5^ Conversely, heart failure and sudden cardiac death are more common in advanced CKD, suggesting that cardiac structural and functional changes (uremic cardiomyopathy) rather than coronary disease may be the mediator of adverse events.^6^ Evidence from echocardiography and cardiac magnetic resonance (CMR) imaging studies suggests that adverse cardiac structural and functional change in CKD including elevated left ventricular (LV) mass begins early in CKD.^7–9^

Studying living kidney donors allows examination of the isolated effects of a reduction in kidney function on the cardiovascular system in healthy subjects. To date, most clinical outcome studies from kidney donors have not demonstrated an increase in major cardiovascular events.^10^ A recent 15-year retrospective study of living kidney donors, however, reported an increase in cardiovascular mortality with a hazard ratio of 1.40 compared with healthy controls raising concern about the long-term safety of kidney donation.^11^ Furthermore, the CRIB (Chronic Renal Impairment in Birmingham)-DONOR study (https://www.clinicaltrials.gov; unique identifier: NCT01028703) highlighted potentially important short-term adverse changes in cardiovascular structure and function.^12^ Compared with controls, donors at 12 months after nephrectomy had an increase in LV mass, deterioration in myocardial strain, and arterial function without change in blood pressure (BP).^12^ The CRIB-DONOR II study was designed to follow-up the same cohort at 5 years to examine the medium-term effects of kidney donation on cardiovascular structure, function, and hemodynamics.

## Methods

### Transparency and Openness Promotion Statement

The data that support the findings of this study are available from the corresponding author upon reasonable request. Preregistration of the study can be found at https://www.clinicaltrials.gov (unique identifier: NCT02973607).

### Study Design and Population

CRIB-DONOR II (NCT02973607) was a longitudinal, 5-year prospective parallel-group study designed to follow-up kidney donors and healthy controls recruited into the CRIB-DONOR study. All participants who originally consented to take part in the CRIB-DONOR study (NCT01028703) were approached for follow-up between May 2017 and May 2019.^12^

### Statement of Ethics

Ethical approval was obtained from the West Midlands Solihull Research Ethics Committee (REC 17/WM/0048) and approved by the Health Research Council. All subjects gave informed consent to take part in accordance with the principles set out in the Declaration of Helsinki.

### Study Protocol

The study was designed to collect data ≈5 years after the original date of enrollment and as far as possible, to use the same methods, equipment, and assays described in the CRIB-DONOR study.^12,13^ The methods and protocol have been published previously.^13^

### CMR Acquisition

CMR studies (3T Magnetom Skyra; Siemens, Germany) were performed at 5 years using the same standard steady-state free-precession cine and aortic distensibility imaging protocol as described previously (Data Supplement, Supplemental Methodology 1).^12^

### CMR Analysis

All LV mass and volume measurements were made at a central CMR core laboratory by 2 independent expert observers (A.M.P. and W.E.M.) blinded to both donor/control status and temporal order (cvi42 software, version 5.3.4; Circle Cardiovascular Imaging, Canada). Delineation of trabeculations and papillary muscles was performed using thresholding to determine the endocardial border.^14^ Papillary muscles were excluded from blood pool volumes and included in calculations of LV mass.^14^ For reproducibility of LV mass methodology, see the Data Supplement (Supplemental Methodology 1). Three-dimensional tissue tracking for 3-dimensional global circumferential strain and global longitudinal strain was performed as previously described with the baseline and 12-month data reanalyzed (TomTec 2D not available for CRIB-DONOR II) to allow comparison.^12,15^ Aortic distensibility was assessed using software developed in Matlab, version R2017a (Mathworks; Data Supplement, Supplemental Methodology 2).^16^

### Assessment of Late Gadolinium Enhancement, T1 and T2 Mapping

Late gadolinium enhancement (LGE) was defined based on definitions described previously.^17^ Quantification was made using full-width half-maximum methodology.^18^ For assessment of T1, T2, and extracellular volume, the myocardium at the midventricular slice was segmented into American Heart Association segments, and global values were calculated as an average of the valid segments (Figure S1 in the Data Supplement).^19–21^

### Noninvasive Measures of Arterial Stiffness

Pulse wave analysis, pulse wave velocity, and central BP were measured with the SphygmoCor device (Atcor Medical, Sydney, Australia) and a high-fidelity micromanometer (SPC-301; Millar Instruments, Houston, TX) as described previously (for details, see the Data Supplement, Supplemental Methodology 3).^13,22,23^

### Blood Pressure

Office BP and heart rate were measured after 15 minutes of supine rest using the BpTRU (BPM_100 model) with an appropriate size cuff on the nondominant arm with the elbow rested on a pillow in the dorsiflex position.^24^ Five readings were recorded at regular intervals over 5 minutes and the mean taken by a trained observer.^25^

At the end of the study, subjects were fitted with ambulatory BP monitors (Mobil-O-Graph; IEM Gmbh, Stolberg, Germany) set to measure BP every 30 minutes during the day (from 8:00 to 22:00) and every hour at night (from 22:01 to 7:59). An appropriate size cuff was chosen and fitted to the nondominant arm by a trained observer. The validity of a recording and definition of hypertension was in accordance with the European Society of Hypertension guidelines.^26^

### Carotid Intima-Media Thickness

Carotid intima-media thickness was measured in real time using ultrasound (Philips iE33, L9-3Mhz linear array transducer) using IMT QLAB (Philips, United Kingdom) software for automated tracking of the wall.^27^ Three measurements were taken 1 cm from the carotid bifurcation, and the mean of both internal carotid arteries was used in final analysis.^12^

### Determination of Kidney Function

Isotopic glomerular filtration rate (iGFR) measurement was determined using clearance of chromium-51 labeled EDTA in accordance with the British Nuclear Medicine Society guidelines.^28^ At 5 years, kidney donors but not controls underwent iGFR assessment. For assessment of isotopic glomerular filtration rate, a total of 1.85 MBq of chromium-51 labeled EDTA was injected into a vein in the antecubital fossa. Venous blood samples were taken at 2, 3, and 4 hours post-injection if the eGFR was >60 mL/min per 1.73 m2, otherwise 2, 4, and 6 hours if <60 mL/min per 1.73 m2. Samples were counted the following day using a Cobra Auto Gamma Counter (Packard, Ltd). The CKD Epidemiology Collaboration 2009 equation was used to calculate eGFR.^29^

### Biochemical Assays

FGF23 (fibroblast growth factor-23) was measured using frozen plasma stored at −80 °C, using the C-terminal kit from Immunotopics (catalog No. 60-6100). N-terminal pro-B-type natriuretic peptide and high-sensitivity troponin T were measured on frozen serum stored at −80 °C, using the Elecsys Cobas immunoassay (Roche Diagnostics).

### Outcome Measures, Sample Size, and Power

The primary end point was change in LV mass at 5 years compared with baseline. Exploratory secondary end points included changes in BP, pulse wave analysis, pulse wave velocity, aortic distensibility, biomarkers, and carotid intima-media thickness. For details of a combined BP end point, see the Data Supplement (Supplemental Methodology 4). Using the effect sizes and variances from the CRIB-DONOR study (change in LV mass, 7 g; SD of change, 10 g), recruiting 50 subjects in each group would provide 93% power to detect a difference in LV mass of 7 g with an alpha value of 0.05.^12,30^ For 80% power, 34 subjects in each group were required.

### Statistical Analysis

Statistical analysis was performed using SPSS, version 23 (IBM, Armonk, NY). Continuous variables were assessed graphically using histograms to determine normality. Nonparametric data were log10 transformed and assessed graphically. For continuous data, within-group change from baseline to 12 months and baseline to 5 years was analyzed using paired samples *t* tests. Between-group difference was analyzed using independent samples *t* tests to compare within-group change at 5 years between groups and generate the *P* for the primary end point. Nonparametric data were analyzed in a logged format and then antilogged and displayed as multipliers. Categorical data are displayed as counts and percentages, between-group changes are displayed as relative risks and 95% CIs, and analyses were performed using MedCalc for Windows, version 19.4 (MedCalc Software, Ostend, Belgium). Interactions between each variable and donor/control status were determined by general linear models. Multivariable model analysis was performed using linear regression and incorporating any significant interactions. An interval-censored cox regression was used for analysis of the combined BP end point using the icenReg package in R. T1, T2, and extracellular volume, which were measured at 5 years only, were analyzed using independent samples *t* tests. Reproducibility was assessed using intraclass correlation coefficients.

## Results

### Study Subjects

**Figure 1.**
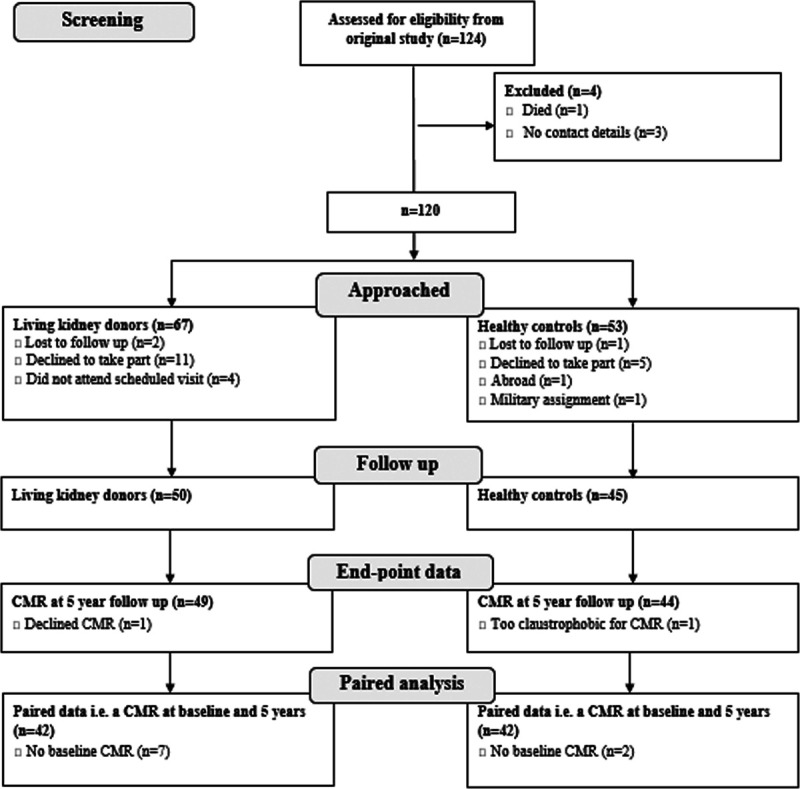
**Flowchart of recruitment.** CMR indicates cardiac magnetic resonance.

Records from all 124 subjects who took part in the original study were reviewed. Of these, 1 had died of bronchial carcinoma, and 3 were not contactable; 120 were approached; 50 kidney donors and 45 healthy controls agreed to participate (Figure 1). There were no significant differences in baseline demographics between those who attended follow-up at 5 years and those who were lost to follow-up other than a cardiovascular family history (for details see Data Supplement, Supplemental Table S1).

One kidney donor and one healthy control declined a CMR study. Nine subjects did not undergo a baseline CMR study; therefore, there were 42 kidney donors and 42 controls with paired sets of end-point data (baseline and 5-year CMR data). Three subjects had contraindications to 3T CMR and had 1.5T scans using the same protocol.

### Subject Characteristics

Data are presented in Table 1. One control subject was diagnosed with diabetes and one with ischemic heart disease. There was an increase from baseline in the prevalence of self-reported hypertension in kidney donors (4%–16%) with little change in controls (7%–9%). At 5 years, the proportion of donors and controls on antihypertensive medication was not different between groups.

**Table 1.**
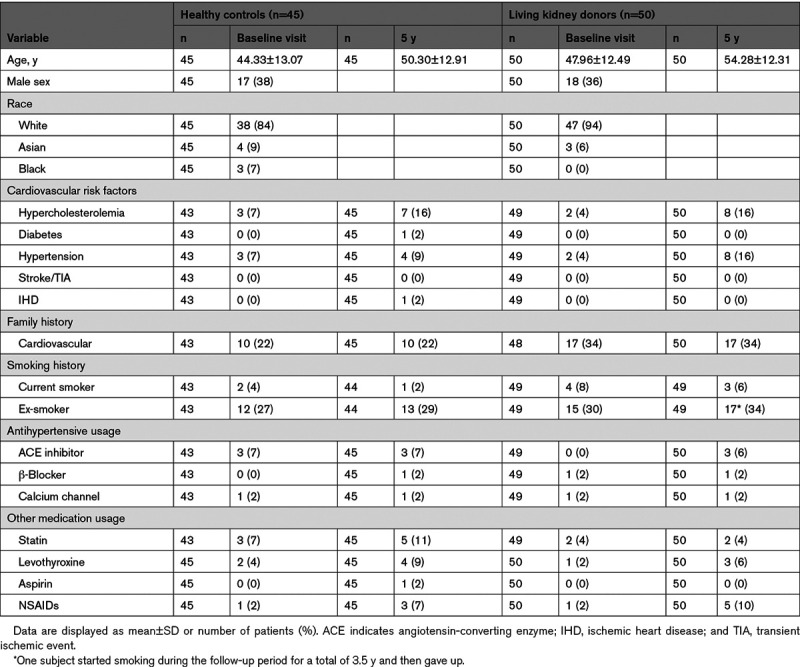
Clinical Demographics at Baseline and 5 y

### Events

There were no deaths or major cardiovascular events in subjects during the study period. For details of all incidental findings during the study, see Data Supplement Supplemental Table S2.

### Kidney Function

In kidney donors, the mean eGFR was 95±15 mL/min per 1.73 m2 at baseline before donation, 65±13 mL/min per 1.73 m2 at 12 months, and 67±14 mL/min per 1.73 m2 at 5 years. Changes in iGFR (normalized to body surface area) in kidney donors were comparable: baseline, 91±12 mL/min per 1.73 m2; 12 months, 59±11 mL/min per 1.73 m2; 5 years, 64±11 mL/min per 1.73 m2. In controls, there was a mean −1±2 mL/min per 1.73 m2 decline annually in eGFR: baseline, 99±16 mL/min per 1.73 m2; 12 months, 96±15 mL/min per 1.73 m^2^; 5 years, 94±15 mL/min per 1.73 m^2^.

### Effects on LV Mass, Volumes, Geometry, and Function

**Figure 2.**
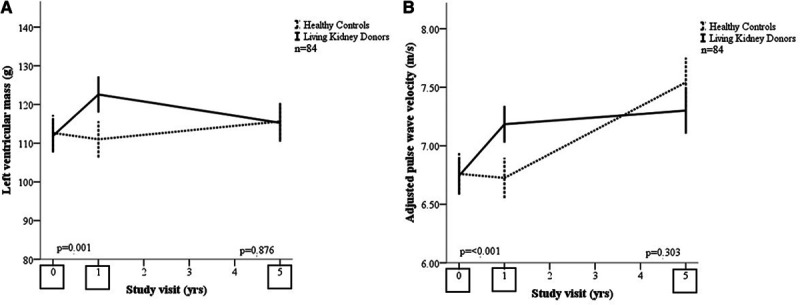
**Longitudinal change in left ventricular mass and pulse wave velocity before and after donation in donors and controls.** Data plotted include data available at baseline and at 5 y. Black solid lines are means with standard errors for donors. Black dashed lines are means and SEs for controls. Black squares indicate study visits. The *P* values are from independent samples *t* tests of the between-group difference for 1- and 5-y change for participants with paired data sets. **A**, Left ventricular mass (g). **B**, Adjusted pulse wave velocity (m/s).

At 5 years, change in LV mass in kidney donors was not different from healthy controls, +0.40 g ([95% CI, −4.68 to 5.49] *P*=0.876) Table 2; Figure 2A.

There was no significant difference in the changes in LV or left atrial volumes indexed for body surface area, LV geometry, global longitudinal strain, or global circumferential strain at 5 years (Table 2).

**Table 2.**
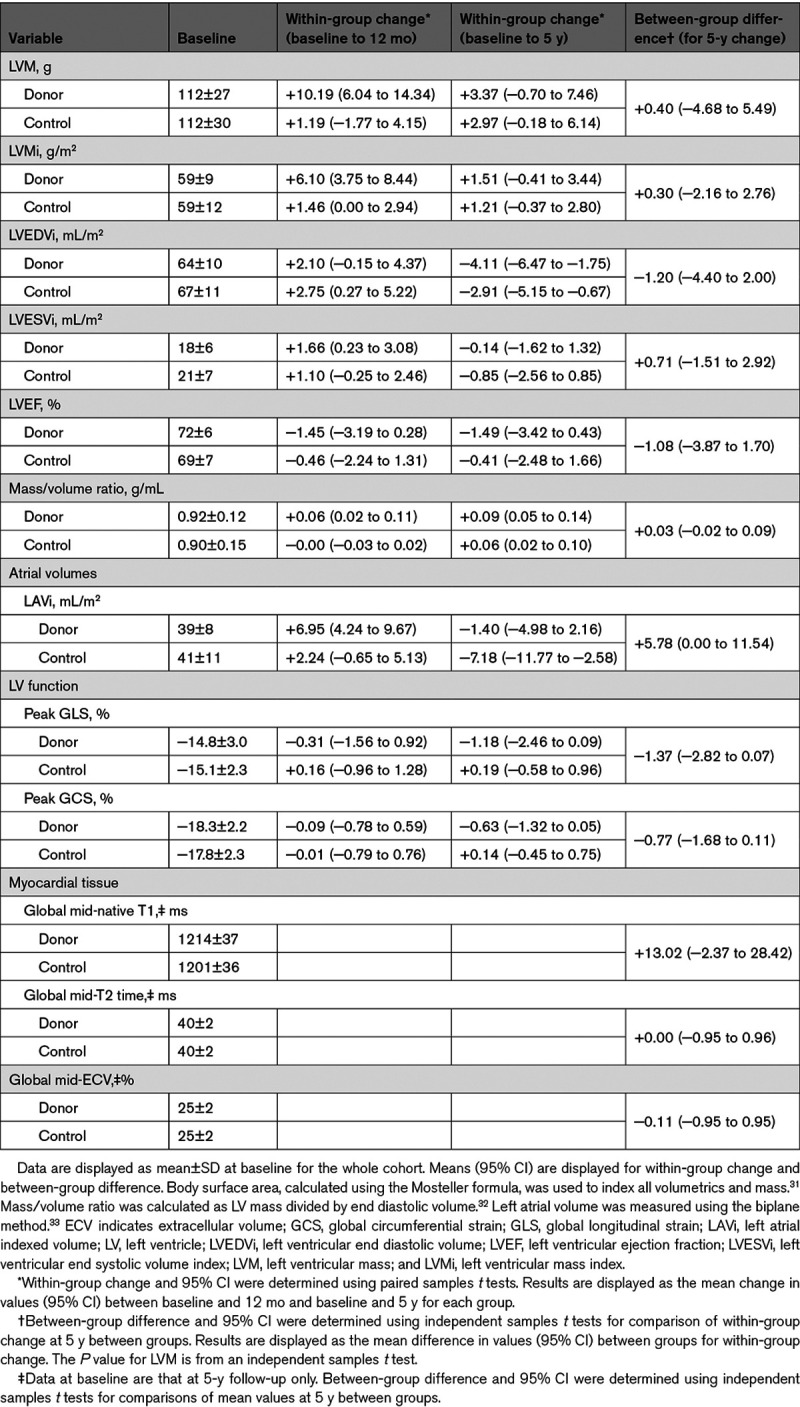
Cardiovascular Structural and Functional Effects

### Myocardial Tissue Characterization

Forty-eight kidney donors and 42 healthy controls underwent 3T T1 and T2 mapping at 5 years. Neither global native T1 time nor T2 time was significantly different in kidney donors compared with controls in the midventricular slice (Table 2). In the 44 kidney donors and 34 controls who consented to contrast, there was no significant difference in mean extracellular volume (Table 2). LGE at the right ventricular insertion points was seen in 4 living kidney donors (percentage of LV mass, 0.87±0.15%) and in one control. There was no LV myocardial LGE.

### Effects on Hemodynamics and Arterial Stiffness and Structure

There were no between-group differences in office BP or heart rate at 5 years (Table 3). Compared with baseline, office systolic BP fell in both groups at 5 years. Ambulatory and central BPs, however, increased in both groups over time but were not significantly different between groups at 5 years. The proportion of subjects with a diagnosis of hypertension (on ambulatory BP monitoring criteria) showed no significant differences (Table 3). A further subanalysis using a composite end point of clinically significant increases in BP also showed no significant differences between the two groups. The hazard ratio for hypertension using the combined outcome in donors relative to controls was increased but not significant (hazard ratio, 1.38 [95% CI, 0.74–2.60]; *P*=0.313; Data Supplement, Supplemental Methodology 4). Carotid intima-media thickness at 5 years was greater in donors versus controls but had not increased significantly from previous values.

**Table 3.**
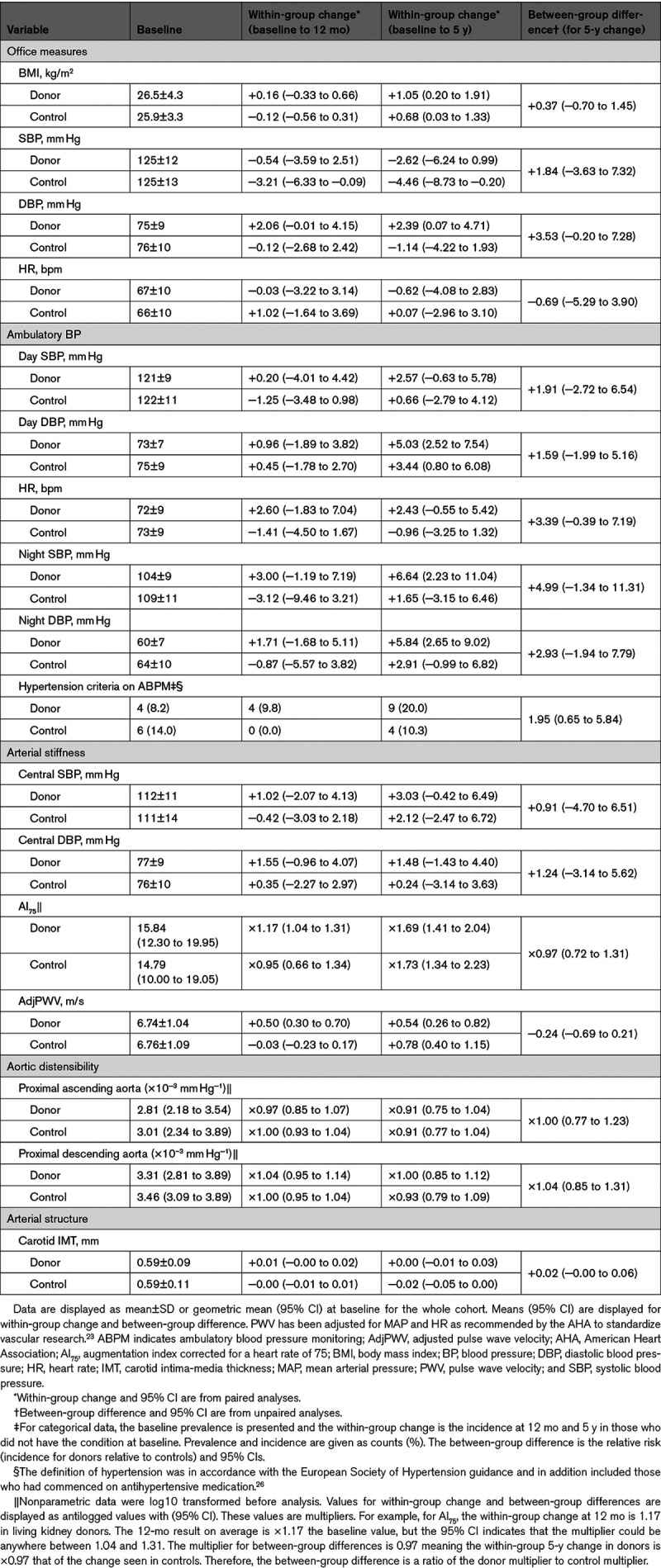
Blood Pressure, Central Hemodynamic and Vascular Effects

At 12 months, there was an increase from baseline in pulse wave velocity in kidney donors, which was not seen in controls. From 12 months to 5 years, the pulse wave velocity increased in both groups, and by 5 years, the between-group difference was not significantly different (Figure 2B). A similar pattern was observed in augmentation index corrected for a heart rate of 75 in which there was a small increase in kidney donors at 12 months compared with a fall in healthy controls. Augmentation index corrected for a heart rate of 75 at 5 years was not significantly different between kidney donors and controls. Aortic distensibility in the proximal ascending and descending aorta decreased in both groups over time with no between-group difference.

### Biochemical Effects

Biochemical data are given in Table S3. There was an increase in high-sensitivity C-reactive protein, high-sensitivity troponin T, and vitamin D over time in both donors and controls. At 12 months, the prevalence of detectable troponin T was greater in donors than controls; at 5 years, the prevalence had increased in both groups reducing the between-group difference.^12^ Serum urate and FGF23 were higher in donors than controls at 5 years.

### Factors Influencing Change in LV Mass

A linear regression analysis was performed to determine variables influencing change in LV mass from baseline to 5 years adjusted for both follow-up time and donor/control status (see Data Supplement, Supplemental Table S4). There was no significant influence of sex or LV mass at baseline on change in LV mass at 5 years. Change in ambulatory systolic BP, however, was significantly associated with change in LV mass. None of the other variables were significant when included in a multivariable model with change in day systolic BP.

### Reproducibility for Primary End Point

There was high reproducibility for LV mass assessment. The interclass correlation coefficients (95% CIs) for interstudy, intraobserver, and interobserver variability were 0.99 (0.98–0.99), 0.99 (0.96–0.99), and 0.99 (0.97–0.99), respectively (see Data Supplement, Supplemental Table S5).

## Discussion

The major findings of this 5-year prospective study of kidney donors were that there were no significant differences compared with controls in LV mass and other parameters of cardiac structure and function and no significant differences in any measure of BP or arterial stiffness. The increase in LV mass that we reported at 12 months had largely resolved by 5 years. Myocardial characterization with gadolinium enhanced CMR, and T1 mapping techniques also demonstrated no significant differences compared with controls. Of the biomarkers, only serum urate and FGF23 remained significantly elevated compared with controls at 5 years. At this time point, despite the falls in eGFR, kidney donors show no evidence of early uremic cardiomyopathy or of the development of hypertension or increased arterial stiffness beyond the changes occurring in controls attributable to aging. These data should be viewed as reassuring findings for those considering kidney donation and for clinicians involved in live donor transplant programs.

In the first CRIB-DONOR study, there was a significant increase in LV mass in kidney donors compared with healthy controls at 12 months.^12^ These results were confirmed by a later small uncontrolled study of 23 kidney donors.^34^ Our latest results suggest that these changes resolve over time. The reasons for these fluctuations are unclear. Effects due to random chance cannot be excluded, but there may have been influences on LV mass at 12 months due to circulating and hemodynamic factors that we either did not measure or were unable to detect. A contributing factor to the reduction in between-group differences at 5 years may have been the reduction over time in the differences in eGFR. In donors, while 12-month iGFR was reduced by about 30 mL/min per 1.73 m2, by 5 years, there was a mean increase from this nadir of 2 mL/min per 1.73 m^2^. In contrast, eGFR in healthy controls declined by about 1 mL/min per 1.73 m^2^ per year. In the first CRIB-DONOR study, we found a significant association between the increase in LV mass and change in iGFR (β=−0.3; R^2^=0.19; *P*<0.001).^12^ Given this, and the strong associations of LV mass with reduced eGFR in community studies, a reduced difference in eGFR might be expected to be associated with a reduced difference in LV mass.^35–37^ Other direct and indirect effects of the nephrectomy surgery on LV mass seem unlikely to explain the 12-month findings. Although donors experience an acute reduction in hemoglobin and a rise in erythropoietin and in C-reactive protein, most of these effects have resolved by 12 months.^38^ Laparoscopic nephrectomy seldom causes long-term pain and is not known to result in autonomic dysfunction. The prevalence of late anemia in kidney donors has been reported at only 11%; consistent with this, we found no difference in hemoglobin at 12 months in our cohort.^39^ We did not, however, measure erythropoietin, which has been associated with LV hypertrophy.^40^

Change in LV mass was chosen as the primary outcome for this study because of the well-recognized association of LV hypertrophy with adverse clinical outcomes and the graded relationship between LV mass and prognosis.^41^ In the Framingham study, LV mass was second only to age in its ability to predict cardiovascular morbidity and mortality.^42^ We acknowledge that a causative relationship cannot be assumed and that a meta-analysis has questioned the validity of using LV mass as a surrogate for total mortality in CKD; this study, however, included patients on dialysis, and many of the studies were of inadequate size and duration and measured LV mass by echocardiography, which has limitations in CKD subjects.^43^

The increase in self-reported hypertension in the living kidney donor group at 5 years was not consistent with the use of antihypertensives or associated with a significant increase in mean office or ambulatory BPs compared with the control group. Likewise, we found no significant difference in hypertension prevalence according to the European Society of Hypertension ambulatory BP monitoring criteria or combined end-point analysis.^26^ It is likely that the apparent finding of increased rates of hypertension in donors was a result of surveillance bias.^44^ This phenomenon has been seen repeatedly in living kidney donor studies.^44^ Our study was not powered to detect small effects on BP, and as the ambulatory BP values in donors at 5 years were numerically slightly higher than those in controls, we suggest that longer and larger studies of ambulatory BP in kidney donors are still required.

This study suggests that a reduction in eGFR of ≈30% as a result of living kidney donation is not inevitably associated with adverse cardiovascular effects including a rise in BP. It is possible that the reduction in eGFR in donors is insufficient to cause cardiovascular damage, but we and others have reported adverse cardiovascular structural and functional findings in subjects with early-stage CKD who have eGFR values similar to our cohort.^45^ Of the donors in our cohort, 36% had a glomerular filtration rate of <60 mL/min per 1.73 m2 at 5 years. The precise threshold at which cardiovascular damage and risk occurs is still a subject under study. Most studies suggest that risk increases at around 60 mL/min per 1.73 m^2^ although effects at levels of kidney function above this have been reported.^46^ It is possible that epidemiological studies have attributed increased cardiovascular risk to early-stage CKD as a result of inadequate correction for traditional risk factors or that factors present in early-stage CKD due to renal injury but not loss of functioning nephrons play a role in the causation of cardiovascular disease. Proteinuria which is commonly viewed as a reflection of inflammatory mediated endothelial damage is a frequent occurrence in early-stage CKD but is seldom seen in donors.^47^ Further long-term studies of cardiovascular disease markers and events in kidney donors are required.

### Strengths and Limitations

The major strength of this study is that it was a blinded end-point analysis from a prospective longitudinal study of a donor cohort with an appropriately healthy control group allowing assessment of serial change. We experienced a high return rate for a longitudinal study with 79% from the original cohort.

Limitations include potential selection bias due to attrition as a result of the longitudinal design. While attempts were made to minimize changes in techniques and methodology, upgrades to our imaging system meant that the magnetic resonance scanner used at 5 years was 3T rather than 1.5T. Signal-to-noise ratio and artifact increase with increasing field strength and can potentially affect scan quality; however, the field strength itself is not deemed to have a significant influence on mass and volume quantification.^48^ Our cohort was predominantly White and, therefore, cannot be generalizable to all kidney donors. It has previously been established that risk is highly likely to be race and age dependent.^44^ Finally, we recognize that due to the large number of variables analyzed, some significant differences are likely to occur by chance and that our sample size limited our ability to detect small changes in secondary end points.

### Perspectives

In summary, we have found no evidence to suggest kidney donation has an adverse effect on cardiovascular structure and function at 5 years over and above those of aging in the general population. The greatest predictor of a change in LV mass in this cohort is in keeping with those well established in the general population, systolic BP.^49^ These results provide reassuring information, suggesting lack of cardiovascular harm and increase in BP at 5 years.

## Acknowledgments

This research was funded by the British Heart Foundation and performed at the National Institute for Health Research (NIHR)/Wellcome Trust Birmingham Clinical Research Facility. The views are those of the authors and not of the National Health Service, the NIHR, or the Department of Health. We would like to thank Peter Nightingale for his statistical input and support.

## Sources of Funding

A.M. Price is supported by a British Heart Foundation Fellowship (FS/16/73/32314). V.M. Stoll is supported by a National Institute for Health Research Clinical Lecturer grant.

## Disclosures

None.

## Supplementary Material



## Novelty and Significance

### What Is New?

- The first serial cardiovascular assessment of donors and healthy controls using advanced magnetic resonance techniques.
- The only description to date of T1, T2, and extracellular volume values in donors compared with healthy controls.

### What Is Relevant?

- Donors and controls have comparable changes in cardiovascular structure and function over time.
- A reduction in glomerular filtration rate after nephrectomy is not inevitably associated with adverse cardiovascular effects.

### Summary

There is no evidence of detrimental changes to cardiovascular structure/function, arterial hemodynamics, blood pressure, or cardiac biomarkers in living kidney donors at 5 years.
