# Supplementary material for: Cardiovascular Effects of Unilateral Nephrectomy in Living Kidney Donors at 5 Years
Source: Hypertension. 2021 Feb 8;77(4):1273–84. doi: 10.1161/HYPERTENSIONAHA.120.15398 (PMC7968960; doi:10.1161/HYPERTENSIONAHA.120.15398)
Supplement: Supplementary file 1 [file hyp-77-1273-s001.pdf]

Cardiovascular Effects of Unilateral Nephrectomy in Living Kidney Donors at Five Years

Short title: Cardiovascular effects of living kidney donation

Anna M. Price[1,2], William E. Moody[3], Victoria M. Stoll[1,3], Ravi Vijapurapu[1,3], Manvir K. Hayer[1,2], Luca Biasiolli[4], Chris J. Weston[5], Rachel Webster[6], Roman Wesolowski[7], Kirsty C. McGee[8], Boyang Liu[1,3], Shanat Baig[1,3], Luke C. Pickup[1,3], Ashwin Radhakrishnan[1,3], Jonathan P. Law[1,2], Nicola C. Edwards[9], Richard P. Steeds[1,3], Charles J. Ferro[1,2], Jonathan N. Townend[1,3]

Corresponding authors details:
Name: Dr Anna M. Price
Address: Cardiology Research Team, Room 19 Clinical Research Offices, Old Nuclear Medicine Department, Queen Elizabeth Hospital, Edgbaston, Birmingham, B15 2TH, United Kingdom
Phone: +44121 371 4624
Email: annaprice@doctors.org.uk

1. Institute of Cardiovascular Sciences, University of Birmingham, Birmingham, UK.
2. University Hospitals NHS Foundation Trust, Department of Nephrology, Birmingham, UK.
3. University Hospitals NHS Foundation Trust, Department of Cardiology, Birmingham, UK
4. Oxford Centre for Magnetic Resonance, University of Oxford, Oxford, UK
5. Institute of Immunology and Immunotherapy, University of Birmingham, Birmingham, UK.
6. University Hospitals NHS Foundation Trust, Department of Biochemistry, Birmingham, UK
7. University Hospitals NHS Foundation Trust, Department of Imaging, Birmingham, UK
8. Institute of Inflammation and Ageing, University of Birmingham, Birmingham, UK.
9. Green Lane Cardiovascular Service, Auckland, NZ

Title: 86/120 characters

Short title: 48/50 characters

Abstract: 246~~225~~/250 words

Total number of figures: 2 figures, 3 tables

Main text (incl ref and fig legends): 5972/6000

References: 49/50

Perspectives: 77/250 words

Novelty and significance: 96 words

Glossary

ACEi; Angiotensin converting enzyme inhibitor.
ABPM; Ambulatory blood pressure monitoring.
AdjPWV; Adjusted pulse wave velocity.
AHA; American Heart Association.
AI_{75}; Augmentation index corrected for a heart rate of 75.
BHF: British Heart Foundation.
BMI; Body mass index.
CI; Confidence intervals.
CMR; Cardiac magnetic resonance.
CKD; Chronic Kidney Disease.
CKD-EPI; Chronic Kidney Disease Epidemiology Collaboration 2009 equation.
^{51}Cr-EDTA; Chromium-51 labelled ethylenediamine tetraacetic acid.
BP; Blood pressure.
DBP; Diastolic blood pressure.
ECV; Extracellular volume.
FGF23; Fibroblast growth factor 23.
eGFR: Estimated glomerular filtration rate.
GFR; Glomerular filtration rate.
GCS; Global circumferential strain.
GLS; Global longitudinal strain.
HR; Heart rate.
Hr; Hour.
IHD; Ischaemic heart disease.
iGFR; Isotopic glomerular filtration rate
IMT; Intima-media thickness.
LA; Left atrial.
LAVi; Left atrial indexed volume.
LGE; Late gadolinium enhancement.
LV; Left ventricular.
LVEDVI; Left ventricular end diastolic volume.
LVESVI; Left ventricular end systolic volume index.
LVSV; Left ventricular stroke volume.
LVEF; Left ventricular ejection fraction.
LVM; Left ventricular mass.
LVMI; Left ventricular mass index.
MAP; Mean arterial pressure.
MWT; Maximum wall thickness.
N; Number of subjects.
NIHR; National Institute for Health Research.
NSAIDS; Non-steroidal anti-inflammatories.
PWA; Pulse wave analysis
PWV; Pulse wave velocity.
REC; Research Ethics Committee
SBP; Systolic blood pressure.
SSFP; Steady-state free precession.
SD; Standard deviation.
TIA; Transient ischaemic event.
T: Tesla.

Abstract

Kidney donation reduces renal function by approximately 30% allowing study of the cardiovascular effects of a reduced estimated glomerular filtration rate without comorbidities. We report five year results of a longitudinal, parallel group, blinded end-point study of living kidney donors (n=50) and healthy controls (n=45). The primary-end point, left ventricular mass, was measured using cardiac magnetic resonance. Secondary end-points, 24 hr ambulatory blood pressure and pulse wave velocity were measured using ~~24 hr ambulatory~~validated blood pressure monitors and the SphygmoCor device~~ respectively~~. Effect sizes were calculated as differences between change from baseline in the donor and control groups ~~minus and the change in the control group~~. ~~Participants underwent office and 24 hr ambulatory blood pressure measures, multi-parametric cardiac magnetic resonance imaging, biomarkers and measurement of arterial stiffness (SphygmoCor).~~

In donors, estimated glomerular filtration rate was 95 ± 15ml/min/1.73m^2 at baseline (pre-donation) and 67 ± 14ml/min/1.73m^2 at 5 years. In controls there was a -1 ±2 ml/min/1.73m^2 decline per annum. Change in left ventricular mass at 5 years was not significantly different between donors and controls, [mean difference +0.40g (95% CI -4.68, 5.49), p=0.876], despite an initial increase in mass in donors compared to controls at 12 months. ~~There was no difference between donors and controls in left ventricular volumes/geometry/function, T1 times or extracellular volume.~~ Pulse wave velocity which~~-~~ increased in donors at 12 months returned to levels not different from controls at 5 years, [mean difference -0.24 m/s (95% CI -0.69, 0.21)]. Change in ambulatory systolic blood pressure ~~[mean difference +1.91mmHg (95% CI -2.72, 6.54)]~~ was not different in donors compared to controls [mean difference +1.91mmHg (95% CI -2.72, 6.54)].~~Office and ambulatory blood pressures in donors did not differ significantly from controls.~~

We found no evidence that the reduction in estimated glomerular filtration rate after kidney donation was associated with a change in left ventricular mass detectable by magnetic resonance imaging at 5 years.

Key words: Arterial stiffness; Cardiovascular; Living kidney donors; Left ventricular mass; Chronic Kidney Disease

Introduction

Chronic kidney disease (CKD) is an independent risk factor for cardiovascular morbidity and mortality.[1] There is an inverse association between estimated glomerular filtration rate (eGFR) and cardiovascular risk.[1] This risk remains elevated even after adjusting for comorbidities such as hypertension and diabetes.[1, 2] Whilst the threshold eGFR at which cardiovascular risk rises is debatable many studies have found that risk increases significantly around 60 ml/min/1.73m^2.[3, 4] Although traditional atherosclerotic risk factors commonly accompany CKD, coronary events account for little of the excess mortality.[5] Conversely, heart failure and sudden cardiac death are more common in advanced CKD, suggesting that cardiac structural and functional changes (uraemic cardiomyopathy) rather than coronary disease may be the mediator of adverse events.[6] Evidence from echocardiography and cardiac magnetic resonance (CMR) imaging studies suggest that adverse cardiac structural and functional change in CKD including elevated left ventricular (LV) mass begin early in CKD.[7-9]

Studying living kidney donors allows examination of the isolated effects of a reduction in kidney function on the cardiovascular system in healthy subjects. To date, most clinical outcome studies from kidney donors have not demonstrated an increase in major cardiovascular events.[10] A recent 15 year retrospective study of living kidney donors however, reported an increase in cardiovascular mortality with a hazard ratio of 1.40 compared to healthy controls raising concern about the long term safety of kidney donation.[11] Furthermore, the Chronic Renal Impairment in Birmingham (CRIB)-DONOR study (NCT01028703) highlighted potentially important short term adverse changes in cardiovascular structure and function.[12] Compared to controls, donors at 12 months after nephrectomy had an increase in LV mass, deterioration in myocardial strain and arterial function without change in blood pressure (BP).[12] The CRIB-DONOR II study was designed

to follow up the same cohort at 5 years to examine the medium term effects of kidney donation on cardiovascular structure, function and hemodynamics.

Methods

Transparency and openness promotion statement

The data that support the findings of this study are available from the corresponding author upon reasonable request. Pre-registration of the study can be found at http://clinical trials.gov (registration no: NCT02973607).

Study design and population

CRIB-DONOR II (NCT02973607) was a longitudinal, 5 year prospective parallel group study designed to follow up kidney donors and healthy controls recruited into the CRIB-DONOR study. All participants who originally consented to take part in the CRIB-DONOR study (NCT01028703) were approached for follow up between May 2017 and May 2019.[12]

Statement of ethics

Ethical approval was obtained from the West Midlands Solihull Research Ethics Committee (REC 17/WM/0048) and approved by the Health Research Council. All subjects gave informed consent to take part in accordance with the principles set out in the Declaration of Helsinki.

Study protocol

The study was designed to collect data approximately 5 years after the original date of enrolment and as far as possible, to use the same methods, equipment and assays described in the CRIB-DONOR study.[12, 13] The methods and protocol have previously been published.[13]

Cardiac magnetic resonance acquisition

Cardiac magnetic resonance (CMR) studies (3T Magnetom, Skyra, Siemens, Germany) were performed at 5 years using the same standard steady-state free precession (SSFP) cine and

aortic distensibility imaging protocol as described previously, see **Supplemental methodology 1**.[12]

CMR analysis

All left ventricular mass and volume measurements were made at a central CMR core laboratory by two independent expert observers (A.M.P and W.E.M) blinded to both donor/control status and temporal order (cvi42® software version 5.3.4, Circle Cardiovascular Imaging, Canada). Delineation of trabeculations and papillary muscles was performed using thresholding to determine the endocardial border.[14] Papillary muscles were excluded from blood pool volumes and included in calculations of LV mass.[14] For reproducibility of left ventricular mass methodology see **Supplemental methodology 1.** Three dimensional (3-D) tissue tracking for 3D global circumferential strain (GCS) and global longitudinal strain (GLS), was performed as previously described with the baseline and 12 month data re-analysed (TomTec 2D not available for CRIB-DONOR II) to allow comparison.[12, 15] Aortic distensibility was assessed using software developed in Matlab version R2017a (Mathworks, USA), see **Supplemental methodology 2**.[16]

Assessment of late gadolinium enhancement, T1 and T2 mapping

Late gadolinium enhancement (LGE) was defined based on definitions previously described.[17] Quantification was made using full width half max methodology.[18] For assessment of T1, T2 and ECV, the myocardium at the mid ventricular slice was segmented into American Heart Association (AHA) segments and global values were calculated as an average of the valid segments see **Figure S1**.[19 20, 21]

Non-invasive measures of arterial stiffness

Pulse wave analysis (PWA), pulse wave velocity (PWV) and central BP were measured with the SphygmoCor device (Atcor Medical, Sydney, Australia) and a high fidelity micromanometer (SPC-301; Millar Instruments, Houston, Texas) as previously described, for details see **Supplemental methodology 3**.[13, 22, 23]

Blood pressure

Office BP and heart rate was measured after 15 minutes of supine rest using the BpTRU^{TM} (BPM_100 model) with an appropriate size cuff on the non-dominant arm with the elbow rested on a pillow in the dorsiflex position.[24] Five readings were recorded at regular intervals over five minutes and the mean taken by a trained observer.[25]

At the end of the study subjects were fitted with ambulatory BP monitors (Mobil-O-Graph; IEM Gmbh, Stolberg, Germany) set to measure blood pressure every 30 minutes during the day (from 8:00-22:00) and every hour at night (from 22:01-7:59). An appropriate size cuff was chosen and fitted to the non-dominant arm by a trained observer. The validity of a recording and definition of hypertension was in accordance with the European Society of Hypertension guidelines.[26]

Carotid intima-media thickness

Carotid intima-media thickness (IMT) was measured in real time using ultrasound (Philips iE33, L9-3Mhz linear array transducer) using IMT QLAB (Philips, UK) software for automated tracking of the wall.[27] Three measurements were taken 1cm from the carotid bifurcation and the mean of both internal carotid arteries were used in final analysis.[12]

Determination of kidney function

iGFR measurement was determined using clearance of ^{51}Cr-EDTA in accordance with British Nuclear Medicine Society guidelines.[28] At 5 years, kidney donors but not controls underwent

iGFR assessment. For assessment of isotopic GFR, a total of 1.85MBq of ^{51}Cr-EDTA was injected into a vein in the antecubital fossa. Venous blood samples were taken at 2, 3 and 4 hours post injection if the eGFR was >60ml/min/1.73m^2 otherwise 2, 4, and 6 hours if less than 60ml/min/1.73m^2. Samples were counted the following day using a Cobra Auto Gamma Counter (Packard Ltd). The Chronic Kidney Disease Epidemiology Collaboration (CKD-EPI) 2009 equation was used to calculate eGFR.[29]

Biochemical assays

Fibroblast growth hormone-23 (FGF-23) was measured using frozen plasma stored at -80°C, using the C-terminal kit from Immunotopics® (cat#60-6100). N-terminal-pro B type natriuretic peptide and high sensitivity Troponin T were measured on frozen serum stored at -80°C, using the Elecsys® Cobas immunoassay (Roche Diagnostics).

Outcome measures, sample size and power

The primary end-point was change in LV mass at 5 years compared to baseline. Exploratory secondary end-points included changes in blood pressure, PWA, PWV, aortic distensibility, biomarkers and carotid IMT. For details of a combined blood pressure endpoint see **Supplemental methodology 4.** Using the effect sizes and variances from the CRIB-DONOR study (change in LV mass 7g, standard deviation of change 10g), recruiting 50 subjects in each group would provide 93% power to detect a difference in LV mass of 7g with an alpha value of 0.05.[12, 30] For 80% power, 34 subjects in each group were required.

Statistical analysis

Statistical analysis was carried out using SPSS©, version 23 (IBM, Armonk, New York, USA). Continuous variables were assessed graphically using histograms to determine normality. Non-parametric data was log10 transformed and assessed graphically. For continuous data, within-

group change from baseline to 12 months and baseline to 5 years was analyzed using paired samples t tests. Between-group difference was analyzed using independent samples t tests to compare within-group change at 5 years between groups and generate the p value for the primary end point. Non-parametric data was analyzed in a logged format then anti-logged and displayed as multipliers. Categorical data are displayed as counts and percentages, between-group changes are displayed as relative risks and 95% confidence intervals and analyses were performed using MedCalc for Windows, version 19.4 (MedCalc Software, Ostend, Belgium). Interactions between each variable and donor/control status were determined by general linear models. Multivariable model analysis was carried out using linear regression and incorporating any significant interactions. An interval censored cox regression was used for analysis of the combined blood pressure endpoint using the icenReg package in R. T1, T2 and ECV, which were measured at 5 years only, were analyzed using independent samples t tests. Reproducibility was assessed using intraclass correlation coefficients.

Results

Study subjects

Records from all 124 subjects who took part in the original study were reviewed. Of these, 1 had died of bronchial carcinoma and 3 were not contactable; 120 were approached; 50 kidney donors and 45 healthy controls agreed to participate, see **Figure 1.** There were no significant differences in baseline demographics between those who attended follow up at 5 years and those that were lost to follow up other than a cardiovascular family history, see **Table S1.**

One kidney donor and one healthy control declined a CMR study. Nine subjects did not undergo a baseline CMR study, therefore, there were 42 kidney donors and 42 controls with paired sets of end-point data (baseline and 5 year CMR data). Three subjects had contraindications to 3T CMR and had 1.5T scans using the same protocol.

Subject characteristics

Data are presented in **Table 1**. One control subject was diagnosed with diabetes and one with ischaemic heart disease. There was an increase from baseline in the prevalence of self-reported hypertension in kidney donors (4% to 16%) with little change in controls (7% to 9%). At 5 years the proportion of donors and controls on anti-hypertensive medication was not different between groups.

Events

There were no deaths or major cardiovascular events in subjects during the study period. For details of all incidental findings during the study, see **Table S2**.

Kidney function

In kidney donors, the mean eGFR was 95 ± 15 ml/min/1.73m^2 at baseline before donation, 65 ±13 ml/min/1.73m^2 at 12 months and 67 ± 14 ml/min/1.73m^2 at 5 years. Changes in iGFR (normalised to body surface area) in kidney donors were comparable: baseline 91 ± 12

ml/min/1.73m^2; 12 months 59 ± 11 ml/min/1.73m^2; 5 years 64 ± 11 ml/min/1.73m^2. In controls there was a mean -1 ± 2ml/min/1.73m^2 decline annually in eGFR: (baseline 99 ± 16 ml/min/1.73m^2; 12 months 96 ± 15 ml/min/1.73m^2; 5 years 94 ± 15 ml/min/1.73m^2).

Effects on LV mass, volumes, geometry and function

At 5 years, change in LV mass in kidney donors were not different to healthy controls +0.40g (95% CI -4.68, 5.49), p=0.876, see **Table 2** and **Figure 2A~~a~~**.

There was no significant difference in the changes in LV or left atrial (LA) volumes indexed for body surface area, LV geometry, GLS or GCS at 5 years, see **Table 2**.

Myocardial tissue characterization

Forty eight kidney donors and 42 healthy controls underwent 3T T1 and T2 mapping at 5 years. Neither global native T1 time nor T2 time was significantly different in kidney donors compared to controls in the mid ventricular slice, see **Table 2**. In the 44 kidney donors and 34 controls who consented to contrast, there was no significant difference in mean ECV, see **Table 2**. LGE at the right ventricular insertion points was seen in 4 living kidney donors (% of LV mass, 0.87± 0.15%) and in one control. There was no left ventricular myocardial LGE.

Effects on hemodynamics and arterial stiffness and structure

There were no between-group difference in office blood pressure or heart rate at 5 years, see **Table 3**. Compared to baseline, office systolic blood pressure fell in both groups at 5 years. Ambulatory and central blood pressures however increased in both groups over time but were not significantly different between-groups at 5 years. The proportion of subjects with a diagnosis of hypertension (on ambulatory blood pressure monitoring criteria) showed no significant differences, see **Table 3**. A further sub analysis using a composite end-point of

clinically significant increases in blood pressure also showed no significant differences between the two groups. The hazard ratio ~~(HR)~~ for hypertension using the combined outcome in donors relative to controls was increased but not significant, [hazard ratio~~HR~~ 1.38 (95% confidence interval 0.74, 2.60), p=0.313], see **Supplemental methodology 4**. Carotid IMT at 5 years was greater in donors vs. controls but had not increased significantly from previous values.

At 12 months, there was an increase from baseline in PWV in kidney donors, which was not seen in controls. From 12 months to 5 years, PWV increased in both groups and by 5 years the between-group difference was not significantly different, see **Figure 2B~~b~~.** A similar pattern was observed in AI_{75} in which there was a small increase in kidney donors at 12 months compared to a fall in healthy controls. AI_{75} at 5 years was not significantly different between kidney donors and controls. Aortic distensibility in the proximal ascending and descending aorta decreased in both groups over time with no between-group difference.

Biochemical effects

Biochemical data are given in **Table S3** There was an increase in high sensitivity C - reactive protein, high sensitivity troponin T and vitamin D over time in both donors and controls. At 12 months, the prevalence of detectable troponin T was greater in donors than controls; at 5 years the prevalence had increased in both groups reducing the between-group difference.[12] Serum urate and FGF23 were higher in donors than controls at 5 years.

Factors influencing change in left ventricular mass

A linear regression analysis was carried out to determine variables influencing change in LV mass from baseline to 5 years adjusted for both follow up time and donor/control status, see **Table S4**. There was no significant influence of sex or LV mass at baseline on change in LV mass at 5 years. Change in ambulatory systolic blood pressure however, was significantly

associated with change in LV mass. None of the other variables were significant when included in a multivariable model with change in day systolic blood pressure.

Reproducibility for primary end-point

There was high reproducibility for LV mass assessment. The interclass correlation coefficients (95% confidence intervals) for inter-study, intra-observer and inter-observer variability were: 0.99 (0.98 - 0.99), 0.99 (0.96 - 0.99) and 0.99 (0.97 - 0.99), respectively (**Table S5)**.

Discussion

The major findings of this 5 year prospective study of kidney donors were that there were no significant differences compared to controls in LV mass and other parameters of cardiac structure and function and no significant differences in any measure of blood pressure or arterial stiffness. The increase in LV mass that we reported at 12 months had largely resolved by 5 years. Myocardial characterization with gadolinium enhanced CMR and T1 mapping techniques also demonstrated no significant differences compared to controls. Of the biomarkers, only serum urate and FGF23 remained significantly elevated compared to controls at 5 years. At this time point, despite the falls in eGFR, kidney donors show no evidence of early uremic cardiomyopathy or of the development of hypertension or increased arterial stiffness beyond the changes occurring in controls attributable to ageing. These data should be viewed as reassuring findings for those considering kidney donation and for clinicians involved in live donor transplant programs.

In the first CRIB-DONOR study, there was a significant increase in LV mass in kidney donors compared to healthy controls at 12 months.[12] These results were confirmed by a later small uncontrolled study of 23 kidney donors.[31] Our latest results suggest that these changes resolve over time. The reasons for these fluctuations are unclear. Effects due to random chance cannot be excluded but there may have been influences on LV mass at 12 months due to circulating and haemodynamic factors which we either did not measure or were unable to detect. A contributing factor to the reduction in between group differences at 5 years may have been the reduction over time in the differences in eGFR. In donors, while 12 month iGFR was reduced by about 30ml/min/1.73m^2, by 5 years there was a mean increase from this nadir of 2ml/min/1.73m^2. In contrast, eGFR in healthy controls declined by about 1 ml/min/1.73m^2 per year. In the first CRIB-DONOR study, we found a significant association between the increase in LV mass and change in iGFR (β=−0.3; R2=0.19; P<0.001).[12] Given

this, and the strong associations of LV mass with reduced eGFR in community studies a reduced difference in eGFR might be expected to be associated with a reduced difference in LV mass.[32-34] Other direct and indirect effects of the nephrectomy surgery on LV mass seem unlikely to explain the 12 month findings. Although donors experience an acute reduction in hemoglobin and a rise in erythropoietin and in C-reactive protein, most of these effects have resolved by 12 months.[35] Laparoscopic nephrectomy seldom causes long term pain and is not known to result in autonomic dysfunction. The prevalence of late anaemia in kidney donors has been reported at only 11%, consistent with this we found no difference in hemoglobin at 12 months in our cohort.[36] We did not however measure erythropoietin, which has been associated with LV hypertrophy.[37]

Change in LV mass was chosen as the primary outcome for this study because of the well-recognised association of LV hypertrophy with adverse clinical outcomes and the graded relationship between LV mass and prognosis.[38] In the Framingham study, LV mass was second only to age in its ability to predict cardiovascular morbidity and mortality.[39] We acknowledge that a causative relationship cannot be assumed and that a meta-analysis has questioned the validity of using LV mass as a surrogate for total mortality in CKD, this study however, included patients on dialysis and many of the studies were of inadequate size and duration and measured LV mass by echocardiography which has limitations in CKD subjects.[40]

The increase in self-reported hypertension in the living kidney donor group at 5 years was not consistent with the use of anti-hypertensives or associated with a significant increase in mean office or ambulatory blood pressures compared to the control group. Likewise, we found no significant difference in hypertension prevalence according to European Society of Hypertension ambulatory blood pressure monitoring criteria or combined endpoint analysis.[26]

It is likely that the apparent finding of increased rates of hypertension in donors was a result of surveillance bias.[41] This phenomenon has been seen repeatedly in living kidney donor studies.[41] Our study was not powered to detect small effects on blood pressure and as the ambulatory blood pressure values in donors at 5 years were numerically slightly higher than those in controls we suggest that longer and larger studies of ambulatory blood pressure in kidney donors are still required.

This study suggests that a reduction in eGFR of approximately 30% as a result of living kidney donation is not inevitably associated with adverse cardiovascular effects including a rise in blood pressure. It is possible that the reduction in eGFR in donors is insufficient to cause cardiovascular damage but we and others have reported adverse cardiovascular structural and functional findings in subjects with early stage CKD who have eGFR values similar to our cohort.[42] Of the donors in our cohort, 36% had a GFR of <60ml/min/1.73m^2 at 5 years. The precise threshold at which cardiovascular damage and risk occurs is still a subject under study. Most studies suggest that risk increases at around 60ml/min/1.73m^2 although effects at levels of kidney function above this have been reported.[43] It is possible that epidemiological studies have attributed increased cardiovascular risk to early stage CKD as a result of inadequate correction for traditional risk factors, or that factors present in early stage CKD due to renal injury but not loss of functioning nephrons play a role in the causation of cardiovascular disease. Proteinuria which is commonly viewed as a reflection of inflammatory mediated endothelial damage, is a frequent occurrence in early stage CKD but is seldom seen in donors.[44] Further long term studies of cardiovascular disease markers and events in kidney donors are required.

Strengths and limitations

The major strength of this study is that it was a blinded end-point analysis from a prospective longitudinal study of a donor cohort with an appropriately healthy control group allowing assessment of serial change. We experienced a high return rate for a longitudinal study with 79% from the original cohort.

Limitations include potential selection bias due to attrition as a result of the longitudinal design. Whilst attempts were made to minimise changes in techniques and methodology, upgrades to our imaging system meant that the magnetic resonance scanner used at 5 years was 3T rather than 1.5T. Signal-to-noise ratio and artefact increases with increasing field strength and can potentially effect scan quality, however the field strength itself is not deemed to have a significant influence on mass and volume quantification.[45] Our cohort were predominantly Caucasian and therefore cannot be generalizable to all kidney donors. It has previously been established that risk is highly likely to be race and age dependent.[41] Finally, we recognise that due to the large number of variables analysed, some significant differences are likely to occur by chance and that our sample size limited our ability to detect small changes in secondary end points.

Perspectives

In summary, we have found no evidence to suggest kidney donation has an adverse effect on cardiovascular structure and function at 5 years over and above those of ageing in the general population. The greatest predictor of a change in LV mass in this cohort are in keeping with those well established in the general population, systolic blood pressure.[46] These results provide reassuring information, suggesting lack of cardiovascular harm and increase in blood pressure at 5 years.

Acknowledgements

The research was funded by the British Heart Foundation (BHF) and carried out at the National Institute for Health Research (NIHR)/Wellcome Trust Birmingham Clinical Research Facility. The views are those of the authors and not of the National Health Service, the NIHR or the Department of Health. We would like to thank Peter Nightingale for his statistical input and support.

Sources of funding

AM Price is supported by a BHF Fellowship (FS/16/73/32314). VM Stoll is supported by a NIHR Clinical Lecturer grant.

Disclosures

No conflicts of interest.

References

1. Matsushita, van der Velde M, Astor BC, Woodward M, Levey AS, de Jong PE, Coresh J, Gansevoort RT. Association of estimated glomerular filtration rate and albuminuria with all-cause and cardiovascular mortality in general population cohorts: A collaborative meta-analysis. *The Lancet*.2010; 375:2073-2081

2. Mahmoodi BK, Matsushita K, Woodward M, Blankestijn PJ, Cirillo M, Ohkubo T, Rossing P, Sarnak MJ, Stengel B, Yamagishi K, Yamashita K, Zhang L, Coresh J, de Jong PE, Astor BC. Associations of kidney disease measures with mortality and end-stage renal disease in individuals with and without hypertension: A meta-analysis. *The Lancet*. 2012;380:1649-1661

3. Go AS, Chertow GM, Fan D, McCulloch CE, Hsu C-y. Chronic kidney disease and the risks of death, cardiovascular events, and hospitalization. *New England Journal of Medicine*. 2004;351:1296-1305

4. Matsushita K, Kwak L, Sang Y, Ballew Shoshana H, Skali H, Shah Amil M, Coresh J, Solomon S. Kidney disease measures and left ventricular structure and function: The atherosclerosis risk in communities study. *Journal of the American Heart Association*. 2017; 6 (9):e006259

5. Gansevoort RT, Correa-Rotter R, Hemmelgarn BR, Jafar TH, Heerspink HJL, Mann JF, Matsushita K, Wen CP. Chronic kidney disease and cardiovascular risk: Epidemiology, mechanisms, and prevention. *The Lancet*. 2013; 382:339-352

6. Banerjee D. Sudden cardiac death in haemodialysis: Clinical epidemiology and mechanisms. *Journal of Electrocardiology*. 2016;49:843-847

7. Mangion K, McDowell K, Mark PB, Rutherford E. Characterizing cardiac involvement in chronic kidney disease using cmr-a systematic review. *Current cardiovascular imaging reports*. 2018;11:2-2

8. Edwards NC, Moody WE, Yuan M, Hayer MK, Ferro CJ, Townend JN, Steeds RP. Diffuse interstitial fibrosis and myocardial dysfunction in early chronic kidney disease. *American Journal of Cardiology*.2015; 115:1311-1317

9. Edwards NC, Moody WE, Chue CD, Ferro CJ, Townend JN, Steeds RP. Defining the natural history of uremic cardiomyopathy in chronic kidney disease: The role of cardiovascular magnetic resonance. *JACC Cardiovasc Imaging*. 2014;7 (7):703-714

10. Garg AX, Meirambayeva A, Huang A, Kim J, Prasad GVR, Knoll G, Boudville N, Lok C, McFarlane P, Karpinski M, Storsley L, Klarenbach S, Lam N, Thomas SM, Dipchand C, Reese P, Doshi M, Gibney E, Taub K, Young A. Cardiovascular disease in kidney donors: Matched cohort study. *BMJ*. 2012;344:e1203

11. Mjoen G, Hallan S, Hartmann A, Foss A, Midtvedt K, Oyen O, Reisater A, Pfeffer P, Jenssen T, Leivestad T, Line P-D, Ovrehus M, Dale DO, Pihlstrom H, Holme I, Dekker FW, Holdaas H. Long-term risks for kidney donors. *Kidney Int*. 2014;86:162-167

12. Moody WE, Ferro CJ, Edwards NC, Chue CD, Lin ELS, Taylor RJ, Cockwell P, Steeds RP, Townend JN. Cardiovascular effects of unilateral nephrectomy in living kidney donors. *Hypertension*. 2016; 67 (2):368-377

13. Moody WE, Tomlinson LA, Ferro CJ, Steeds RP, Mark PB, Zehnder D, Tomson CR, Cockcroft JR, Wilkinson IB, Townend JN. Effect of a reduction in glomerular filtration rate after nephrectomy on arterial stiffness and central hemodynamics: Rationale and design of the earnest study(). *American Heart Journal*. 2014;167:141-149.e142

14. Maceira AM, Prasad SK, Khan M, Pennell DJ. Normalized left ventricular systolic and diastolic function by steady state free precession cardiovascular magnetic resonance. *Journal of Cardiovascular Magnetic Resonance*. 2006;8:417-426

15. Liu B, Dardeer AM, Moody WE, Hayer MK, Baig S, Price AM, Leyva F, Edwards NC, Steeds RP. Reference ranges for three-dimensional feature tracking cardiac magnetic resonance: Comparison with two-dimensional methodology and relevance of age and gender. *The International Journal of Cardiovascular Imaging*. 2018;34:761-775

16. Biasiolli L, Hann E, Lukaschuk E, Carapella V, Paiva JM, Aung N, Rayner JJ, Werys K, Fung K, Puchta H, Sanghvi MM, Moon NO, Thomson RJ, Thomas KE, Robson MD, Grau V, Petersen SE, Neubauer S, Piechnik SK. Automated localization and quality control of the aorta in cine cmr can significantly accelerate processing of the uk biobank population data. *PLOS ONE*. 2019;14:e0212272

17. Price AM, Hayer MK, Vijapurapu R, Fyyaz SA, Moody WE, Ferro CJ, Townend JN, Steeds RP, Edwards NC. Myocardial characterization in pre-dialysis chronic kidney disease: A study of prevalence, patterns and outcomes. *BMC Cardiovasc Disord*. 2019;19:295

18. Flett AS, Hasleton J, Cook C, Hausenloy D, Quarta G, Ariti C, Muthurangu V, Moon JC. Evaluation of techniques for the quantification of myocardial scar of differing etiology using cardiac magnetic resonance. *JACC Cardiovasc Imaging*. 2011;4 (2);150-156

19. Cerqueira Manuel D, Weissman Neil J, Dilsizian V, Jacobs Alice K, Kaul S, Laskey Warren K, Pennell Dudley J, Rumberger John A, Ryan T, Verani Mario S. Standardized myocardial segmentation and nomenclature for tomographic imaging of the heart. *Circulation*. 2002;105:539-542

20. Kim PK, Hong YJ, Im DJ, Suh YJ, Park CH, Kim JY, Chang S, Lee H-J, Hur J, Kim YJ, Choi BW. Myocardial t1 and t2 mapping: Techniques and clinical applications. *Korean Journal of Radiology*. 2017;18:113-131

21. Bull S, White S, Piechnik S, Flett A, Ferreira V, Loudon M, Francis J, Karamitsos T, Prendergast B, Robson M, Neubauer S, Moon J, Myerson S. Human non-contrast t1 values and correlation with histology in diffuse fibrosis. *Heart*. 2013;99:932 - 937

22. Savage MT, Ferro CJ, Pinder SJ, Tomson CRV. Reproducibility of derived central arterial waveforms in patients with chronic renal failure. *Clinical Science*. 2002;103:59-65

23. Townsend RR, Wilkinson IB, Schiffrin EL, Avolio AP, Chirinos JA, Cockcroft JR, Heffernan KS, Lakatta EG, McEniery CM, Mitchell GF, Najjar SS, Nichols WW, Urbina EM, Weber T. Recommendations for improving and standardizing vascular research on arterial stiffness. *Hypertension*. 2015;66:698-722

24. Mattu GS, Heran BS, Wright JM. Overall accuracy of the bptru™—an automated electronic blood pressure device. *Blood Pressure Monitoring*. 2004;9:47-52

25. Beckett L, Godwin M. The bptru automatic blood pressure monitor compared to 24 hour ambulatory blood pressure monitoring in the assessment of blood pressure in patients with hypertension. *BMC Cardiovasc Disord*. 2005;5:18-18

26. Parati G, Stergiou G, O'Brien E, Asmar R, Beilin L, Bilo G, Clement D, de la Sierra A, de Leeuw P, Dolan E, Fagard R, Graves J, Head GA, Imai Y, Kario K, Lurbe E, Mallion J-M, Mancia G, Mengden T, Myers M, Ogedegbe G, Ohkubo T, Omboni S, Palatini P, Redon J, Ruilope LM, Shennan A, Staessen JA, vanMontfrans G, Verdecchia P, Waeber B, Wang J, Zanchetti A, Zhang Y. European society of hypertension practice guidelines for ambulatory blood pressure monitoring. *Journal of Hypertension*. 2014;32:1359-1366

27. Touboul PJ, Hennerici MG, Meairs S, Adams H, Amarenco P, Bornstein N, Csiba L, Desvarieux M, Ebrahim S, Hernandez RH, Jaff M, Kownator S, Naqvi T, Prati P, Rundek T, Sitzer M, Schminke U, Tardif JC, Taylor A, Vicaut E, Woo KS.

Mannheim carotid intima-media thickness and plaque consensus (2004–2006–2011): An update on behalf of the advisory board of the 3rd and 4th watching the risk symposium 13th and 15th european stroke conferences, mannheim, germany, 2004, and brussels, belgium, 2006. *Cerebrovascular diseases (Basel, Switzerland)*. 2012;34:290-296

28. Mårtensson J, Groth S, Rehling M, Gref M. Chromium-51-edta clearance in adults with a single-plasma sample. *Journal of Nuclear Medicine*. 1998;39:2131-2137
29. Levey AS, Stevens LA, Schmid CH, Zhang Y, Castro AF, Feldman HI, Kusek JW, Eggers P, Van Lente F, Greene T, Coresh J. A new equation to estimate glomerular filtration rate. *Annals of internal medicine*. 2009;150:604-612
30. Lenth RV. Java applets for power and sample size [computer software]. (2006-2009) Retrieved *January, 1st, 2016* from http://www.stat.uiowa.edu/~rlenth/Power.
31. Altmann U, Böger CA, Farkas S, Mack M, Luchner A, Hamer OW, Zeman F, Debl K, Fellner C, Jungbauer C, Banas B, Buchner S. Effects of reduced kidney function because of living kidney donation on left ventricular mass. *Hypertension*. 2017; 69(2):297-303
32. Bouzas-Mosquera A, Broullón FJ, Álvarez-García N, Peteiro J, Mosquera VX, Castro-Beiras A. Association of left ventricular mass with all-cause mortality, myocardial infarction and stroke. *PloS one*. 2012;7:e45570-e45570
33. Verdecchia P, Angeli F, Borgioni C, Gattobigio R, de Simone G, Devereux RB, Porcellati C. Changes in cardiovascular risk by reduction of left ventricular mass in hypertension: A meta-analysis. *American Journal of Hypertension*. 2003;16:895-899
34. London GM, Pannier B, Guerin AP, Blacher J, Marchais SJ, Darne B. Alterations of left ventricular hypertrophy in and survival of patients receiving hemodialysis: Follow-up of an interventional study. *J Am Soc Nephrol*. 2001;12(12):2759-2767

35. Hampel DJ, Pratschke J, May G, Reinke P, Schindler R. Living kidney donation: Anemia and inflammation in the early postoperative period. *Transplantation Proceedings*. 2006;38:661-663

36. Shah S, Lankowsky B, Gao T, Zaky Z, Stephany BR, Poggio ED. Postdonation anemia in living kidney donors. *Transplantation Proceedings*. 2017;49:1733-1738

37. Ayus JC, Go AS, Valderrabano F, Verde E, de Vinuesa SG, Achinger SG, Lorenzo V, Arieff AI, Luao J. Effects of erythropoietin on left ventricular hypertrophy in adults with severe chronic renal failure and hemoglobin <10 g/dl. *Kidney International*. 2005;68:788-795

38. Schillaci G, Verdecchia P, Porcellati C, Cuccurullo O, Cosco C, Perticone F. Continuous relation between left ventricular mass and cardiovascular risk in essential hypertension. *Hypertension*. 2000;35:580-586

39. Levy D, Garrison RJ, Savage DD, Kannel WB, Castelli WP. Prognostic implications of echocardiographically determined left ventricular mass in the framingham heart study. *New England Journal of Medicine*. 1990;322:1561-1566

40. Badve SV, Palmer SC, Strippoli GFM, Roberts MA, Teixeira-Pinto A, Boudville N, Cass A, Hawley CM, Hiremath SS, Pascoe EM, Perkovic V, Whalley GA, Craig JC, Johnson DW. The validity of left ventricular mass as a surrogate end point for all-cause and cardiovascular mortality outcomes in people with ckd: A systematic review and meta-analysis. *Am J Kidney Dis*. 2016;68:554-563

41. Price AM, Edwards NC, Hayer MK, Moody WE, Steeds RP, Ferro CJ, Townend JN. Chronic kidney disease as a cardiovascular risk factor: Lessons from kidney donors. *Journal of the American Society of Hypertension*. 2018;12:497-505.e494

42. Edwards NC, Hirth A, Ferro CJ, Townend JN, Steeds RP. Subclinical abnormalities of left ventricular myocardial deformation in early-stage chronic kidney disease: The

precursor of uremic cardiomyopathy? *Journal of the American Society of Echocardiography*.2008; 21:1293-1298

43. Van Biesen W, De Bacquer D, Verbeke F, Delanghe J, Lameire N, Vanholder R. The glomerular filtration rate in an apparently healthy population and its relation with cardiovascular mortality during 10 years. *Eur Heart J*. 2007;28:478-483

44. Seliger SL, Salimi S, Pierre V, Giffuni J, Katzel L, Parsa A. Microvascular endothelial dysfunction is associated with albuminuria and ckd in older adults. *BMC nephrology*. 2016;17:82-82

45. Hudsmith LE, Petersen SE, Tyler DJ, Francis JM, Cheng ASH, Clarke K, Selvanayagam JB, Robson MD, Neubauer S. Determination of cardiac volumes and mass with flash and ssfp cine sequences at 1.5 vs. 3 tesla: A validation study. *Journal of Magnetic Resonance Imaging*. 2006;24:312-318

46. Gidding SS, Liu K, Colangelo LA, Cook NL, Goff DC, Glasser SP, Gardin JM, Lima JA. Longitudinal determinants of left ventricular mass and geometry: The coronary artery risk development in young adults (cardia) study. *Circ Cardiovasc Imaging*. 2013;6:769-775

47. Simplified calculation of body-surface area. *New England Journal of Medicine*. 1987;317:1098-1098

48. Bluemke DA, Kronmal RA, Lima JAC, Liu K, Olson J, Burke GL, Folsom AR. The relationship of left ventricular mass and geometry to incident cardiovascular events. *Journal of the American College of Cardiology*. 2008;52:2148

49. Hudsmith LE, Cheng ASH, Tyler DJ, Shirodaria C, Lee J, Petersen SE, Francis JM, Clarke K, Robson MD, Neubauer S. Assessment of left atrial volumes at 1.5 tesla and 3 tesla using flash and ssfp cine imaging. *Journal of Cardiovascular Magnetic Resonance*. 2007;9:673-679

Novelty and Significance

1) What Is New?

- The first serial cardiovascular assessment of donors and healthy controls using advanced magnetic resonance techniques.
- The only description to date of T1, T2 and ECV values in donors compared to healthy controls.

2) What Is Relevant?

- Donors and controls have comparable changes in cardiovascular structure and function over time.
- A reduction in GFR after nephrectomy is not inevitably associated with adverse cardiovascular effects.

3) Summary

There is no evidence of detrimental changes to cardiovascular structure/function, arterial hemodynamics, blood pressure or cardiac biomarkers in living kidney donors at 5 years.

Figure 1; Flow diagram of recruitment.

Figure 2; Longitudinal change in left ventricular mass and pulse wave velocity before and after donation in donors and controls.

Data plotted includes data available at baseline and 5 yearsall avaliable data for 95 participants. Black solid lines are means with standard errors for donors. Black dashed lines are means and standard errors for controls. Black squares indicate study visits. The p values are from independent samples *t* tests of the between-group difference for 1 year change and 5 year change for participants with paired data sets.

A; Left ventricular mass (g) and B; Adjusted pulse wave velocity (m/s).

Table 1; Clinical demographics at baseline and 5 years.

Variable	Healthy controls n=45				Living kidney donors n=50			
	**N**	**Baseline visit**	**N**	**5 years**	**N**	**Baseline visit**	**N**	**5 years**
**Age** (years)	45	44.33 ± 13.07	45	50.30 ± 12.91	50	47.96 ± 12.49	50	54.28 ± 12.31
**Male sex**	45	17 (38)			50	18 (36)		
**Race**								
White	45	38 (84)			50	47 (94)		
Asian	45	4 (9)			50	3 (6)		
Black	45	3 (7)			50	0 (0)		
**Cardiovascular risk factors**								
Hypercholesterolemia	43	3 (7)	45	7 (16)	49	2 (4)	50	8 (16)
Diabetes	43	0 (0)	45	1 (2)	49	0 (0)	50	0 (0)
Hypertension	43	3 (7)	45	4 (9)	49	2 (4)	50	8 (16)
Stroke/TIA	43	0 (0)	45	0 (0)	49	0 (0)	50	0 (0)
IHD	43	0 (0)	45	1 (2)	49	0 (0)	50	0 (0)
**Family history**								

Cardiovascular	43	10 (22)	45	10 (22)	48	17 (34)	50	17 (34)
**Smoking history**								
Current smoker	43	2 (4)	44	1 (2)	49	4 (8)	49	3 (6)
Ex-smoker	43	12 (27)	44	13 (29)	49	15 (30)	49	17* (34)
**Antihypertensive usage**								
ACEi	43	3 (7)	45	3 (7)	49	0 (0)	50	3 (6)
β blocker	43	0 (0)	45	1 (2)	49	1 (2)	50	1 (2)
Calcium channel	43	1 (2)	45	1 (2)	49	1 (2)	50	1 (2)
**Other medication usage**								
Statin	43	3 (7)	45	5 (11)	49	2 (4)	50	2 (4)
Levothyroxine	45	2 (4)	45	4 (9)	50	1 (2)	50	3 (6)
Aspirin	45	0 (0)	45	1 (2)	50	0 (0)	50	0 (0)
NSAIDs	45	1 (2)	45	3 (7)	50	1 (2)	50	5 (10)

Data are displayed as mean ± SD~~, median [IQR]~~ or number of patients (percentage).

ACEi; Angiotensin converting enzyme inhibitor, BMI; Body mass index. IHD; Ischaemic heart disease. N; Number of subjects. NSAIDS; Non-steroidal anti-inflammatories. SD; Standard deviation. TIA; Transient ischaemic event

*One subject started smoking during follow up period for a total of 3.5 years and then gave up.

Table 2; Cardiovascular structural and functional effects.

Variable	Baseline	Within-group change * (baseline to 12 months)	Within-group change * (baseline to 5 years)	Between-group difference † (for 5 year change)
LVM (g)				
Donor	112 ± 27	+10.19 (6.04, 14.34)	+3.37 (-0.70, 7.46)	+0.40 (-4.68, 5.49)
Control	112 ± 30	+1.19 (-1.77, 4.15)	+2.97 (-0.18, 6.14)	
LVMi (g/m^2)				
Donor	59 ± 9	+6.10 (3.75, 8.44)	+1.51 (-0.41, 3.44)	+0.30 (-2.16, 2.76)
Control	59 ± 12	+1.46 (0.00, 2.94)	+1.21 (-0.37, 2.80)	
LVEDVi (ml/m^2)				
Donor	64 ± 10	+2.10 (-0.15, 4.37)	-4.11 (-6.47, -1.75)	-1.20 (-4.40, 2,00)
Control	67 ± 11	+2.75 (0.27, 5.22)	-2.91 (-5.15, -0.67)	

LVESVi (ml/m^2)				
Donor	18 ± 6	+1.66 (0.23, 3.08)	-0.14 (-1.62, 1.32)	+0.71 (-1.51, 2.92)
Control	21 ± 7	+1.10 (-0.25, 2.46)	-0.85 (-2.56, 0.85)	
LVEF (%)				
Donor	72 ± 6	-1.45 (-3.19, 0.28)	-1.49 (-3.42, 0.43)	-1.08 (-3.87, 1.70)
Control	69 ± 7	-0.46 (-2.24, 1.31)	-0.41 (-2.48, 1.66)	
Mass/volume ratio (g/ml)				
Donor	0.92 ± 0.12	+0.06 (0.02, 0.11)	+0.09 (0.05, 0.14)	+0.03 (-0.02, 0.09)
Control	0.90 ± 0.15	-0.00 (-0.03, 0.02)	+0.06 (0.02, 0.10)	
Atrial volumes				
LAVi (ml/m^2)				

Donor	39 ± 8	+6.95 (4.24, 9.67)	-1.40 (-4.98, 2.16)	+5.78 (0.00, 11.54)
Control	41 ± 11	+2.24 (-0.65, 5.13)	-7.18 (-11.77, -2.58)	
LV function				
Peak GLS %				
Donor	-14.8 ± 3.0	-0.31 (-1.56, 0.92)	-1.18 (-2.46, 0.09)	-1.37 (-2.82, 0.07)
Control	-15.1 ± 2.3	+0.16 (-0.96, 1.28)	+0.19 (-0.58, 0.96)	
Peak GCS (%)				
Donor	-18.3 ± 2.2	-0.09 (-0.78, 0.59)	-0.63 (-1.32, 0.05)	-0.77 (-1.68, 0.11)
Control	-17.8 ± 2.3	-0.01 (-0.79, 0.76)	+0.14 (-0.45, 0.75)	
Myocardial tissue				
Global mid native T1 (ms) ‡				
Donor	1214 ± 37			

				+13.02 (-2.37, 28.42)
Control	1201 ± 36			
Global mid T2 time (ms) ‡				
Donor	40 ± 2			+0.00 (-0.95, 0.96)
Control	40 ± 2			
Global mid ECV (%) ‡				
Donor	25 ± 2			-0.11 (-0.95, 0.95)
Control	25 ± 2			

Data are displayed as mean ± SD at baseline for the whole cohort. Mean (95% CI) are displayed for within-group change and between-group difference.

*Within-group change and 95% CI were determined using paired samples *t* tests. Results are displayed as the mean change in values (95% CI) between baseline and 12 months and baseline and 5 years for each group.

† Between-group difference and 95% CI were determined using independent samples *t* tests for comparison of within-group change at 5 years between groups. Results are displayed as the mean difference in values (95% CI) between groups for within-group change. The p value for LVM is from an independent samples *t* test.

‡ Data at baseline is that at 5 year follow up only. Between-group difference and 95% CI were determined using independent samples *t* tests for comparisons of mean values at 5 years between groups.

Body surface area, calculated using the Mosteller formula was used to index all volumetrics and mass.[47] Mass/volume ratio was calculated as left ventricular mass divided by end diastolic volume.[48] Left atrial volume was measured using the biplane method.[49]

CI; Confidence intervals, ECV; Extracellular volume GCS; Global circumferential strain, GLS; Global longitudinal strain, ~~GRS; Global radial strain,~~ LAVi; Left atrial indexed volume. LVEDVI; Left ventricular end diastolic volume. LVESVI; Left ventricular end systolic volume index. LVSV; Left ventricular stroke volume. LVEF; Left ventricular ejection fraction. LVM; Left ventricular mass. LVMI; Left ventricular mass index. MWT; maximum wall thickness. SD; Standard deviation.

Table 3; Blood pressure, central hemodynamic and vascular effects.

**Variable**	**Baseline**	**Within-group change * (baseline to 12 months)**	**Within-group change * (baseline to 5 years)**	**Between-group difference † (for 5 year change)**
Office measures				
BMI (kg/m^2)				
Donor	26.5 ± 4.3	+0.16 (-0.33, 0.66)	+1.05 (0.20, 1.91)	+0.37 (-0.70, 1.45)
Control	25.9 ± 3.3	-0.12 (-0.56, 0.31)	+0.68 (0.03, 1.33)	
SBP (mmHg)				
Donor	125 ± 12	-0.54 (-3.59, 2.51)	-2.62 (-6.24, 0.99)	+1.84 (-3.63, 7.32)
Control	125 ± 13	-3.21 (-6.33, -0.09)	-4.46 (-8.73, -0.20)	
DBP (mmHg)				
Donor	75 ± 9	+2.06 (-0.01, 4.15)	+2.39 (0.07, 4.71)	

				+3.53 (-0.20, 7.28)
Control	76 ± 10	-0.12 (-2.68, 2.42)	-1.14 (-4.22, 1.93)	
HR (bpm)				
Donor	67 ± 10	-0.03 (-3.22, 3.14)	-0.62 (-4.08, 2.83)	-0.69 (-5.29, 3.90)
Control	66 ± 10	+1.02 (-1.64, 3.69)	+0.07 (-2.96, 3.10)	
Ambulatory BP				
Day SBP (mmHg)				
Donor	121 ± 9	+0.20 (-4.01, 4.42)	+2.57 (-0.63, 5.78)	+1.91 (-2.72, 6.54)
Control	122 ± 11	-1.25 (-3.48, 0.98)	+0.66 (-2.79, 4.12)	
Day DBP (mmHg)				
Donor	73 ± 7	+0.96 (-1.89, 3.82)	+5.03 (2.52, 7.54)	+1.59 (-1.99, 5.16)
Control	75 ± 9	+0.45 (-1.78, 2.70)	+3.44 (0.80, 6.08)	

HR (bpm)				
Donor	72 ± 9	+2.60 (-1.83, 7.04)	+2.43 (-0.55, 5.42)	+3.39 (-0.39, 7.19)
Control	73 ± 9	-1.41 (-4.50, 1.67)	-0.96 (-3.25, 1.32)	
Night SBP (mmHg)				
Donor	104 ± 9	+3.00 (-1.19, 7.19)	+6.64 (2.23, 11.04)	+4.99 (-1.34, 11.31)
Control	109 ± 11	-3.12 (-9.46, 3.21)	+1.65 (-3.15, 6.46)	
Night DBP (mmHg)				
Donor	60 ± 7	+1.71 (-1.68, 5.11)	+5.84 (2.65, 9.02)	+2.93 (-1.94, 7.79)
Control	64 ± 10	-0.87 (-5.57, 3.82)	+2.91 (-0.99, 6.82)	
Hypertension criteria on ABPM ‖ ¶				
Donor	4 (8.2)	4 (9.8)	9 (20.0)	1.95 (0.65, 5.84)

Control	6 (14.0)	0 (0.0)	4 (10.3)	
Arterial stiffness				
Central SBP (mmHg)				
Donor	112 ± 11	+1.02 (-2.07, 4.13)	+3.03 (-0.42, 6.49)	+0.91 (-4.70, 6.51)
Control	111 ± 14	-0.42 (-3.03, 2.18)	+2.12 (-2.47, 6.72)	
Central DBP (mmHg)				
Donor	77 ± 9	+1.55 (-0.96, 4.07)	+1.48 (-1.43, 4.40)	+1.24 (-3.14, 5.62)
Control	76 ± 10	+0.35 (-2.27, 2.97)	+0.24 (-3.14, 3.63)	
AI_{75} §				
Donor	15.84 [12.30, 19.95]	×1.17 (1.04, 1.31)	×1.69 (1.41, 2.04)	×0.97 (0.72, 1.31)
Control	14.79 [10.00, 19.05]	×0.95 (0.66, 1.34)	×1.73 (1.34, 2.23)	
Adj PWV (m/s)				

Donor	6.74 ± 1.04	+0.50 (0.30, 0.70)	+0.54 (0.26, 0.82)	-0.24 (-0.69, 0.21)
Control	6.76 ± 1.09	-0.03 (-0.23, 0.17)	+0.78 (0.40, 1.15)	
Aortic distensibility				
Proximal ascending aorta (×10^{-3} mm Hg^{-1}) §				
Donor	2.81 [2.18, 3.54]	×0.97 (0.85, 1.07)	×0.91 (0.75, 1.04)	×1.00 (0.77, 1.23)
Control	3.01 [2.34, 3.89]	×1.00 (0.93, 1.04)	×0.91 (0.77, 1.04)	
Proximal descending aorta (×10–3 mm Hg−1) §				
Donor	3.31 [2.81, 3.89]	×1.04 (0.95, 1.14)	×1.00 (0.85, 1.12)	×1.04 (0.85, 1.31)
Control	3.46 [3.09, 3.89]	×1.00 (0.95, 1.04)	×0.93 (0.79, 1.09)	
Arterial structure				
Carotid IMT~~CIT~~ (mm)				

Donor	0.59 ± 0.09	+0.01 (-0.00, 0.02)	+0.00 (-0.01, 0.03)	+0.02 (-0.00, 0.06)
Control	0.59 ± 0.11	-0.00 (-0.01, 0.01)	-0.02 (-0.05, 0.00)	

Data are displayed as mean ± SD or geometric mean [95% CI] at baseline for the whole cohort. Mean (95% CI) are displayed for within-group change and between-group difference.

*Within-group change and 95% CI are from paired analyses.

† Between-group difference and 95% CI are from unpaired analyses.

§ Non-parametric data was log10 transformed prior to analysis. Values for within-group change and between group differences are displayed as antilogged values with (95% CI). These values are multipliers. For example, for AI[75] the within group change at 12 months is 1.17 in living kidney donors. The 12 month result on average is ×1.17 the baseline value but the 95% confidence interval indicates that the multiplier could be anywhere between 1.04 and 1.31. The multiplier for between group differences is 0.97 meaning the within-group 5 yr. change in donors is ×0.97 that of the change seen in controls. Therefore the between group difference is a ratio of the donor multiplier to control multiplier.

|| For categorical data the baseline prevalence is presented and the within group change is the incidence at 12 months and 5 years in those who did not have the condition at baseline. Prevalence and incidence are given as counts (percentage). The between-group difference is the relative risk (incidence for donors relative to controls) and 95% confidence intervals.

¶ The definition of hypertension was in accordance with the European Society of Hypertension guidance and in addition included those who had commenced on antihypertensive medication.[26]

PWV has been adjusted for MAP and HR as recommended by the AHA to standardise vascular research.[23]

AHA; American Heart Association. ABPM; Ambulatory blood pressure monitoring. AI_{75}; Augmentation index corrected for a heart rate of 75. AdjPWV; Adjusted pulse wave velocity. BMI; Body mass index. CIT; Carotid intima-media thickness. CI; Confidence interval. DBP; Diastolic blood pressure. HR; Heart rate. IMT; Carotid intima-media thickness MAP; Mean arterial pressure. PWV; Pulse wave velocity. SBP; Systolic blood pressure. SD; Standard deviation
