# Supplementary material for: Cardiovascular Effects of Unilateral Nephrectomy in Living Kidney Donors at 5 Years
Source: Hypertension. 2021 Feb 8;77(4):1273–84. doi: 10.1161/HYPERTENSIONAHA.120.15398 (PMC7968960; doi:10.1161/HYPERTENSIONAHA.120.15398)
Supplement: Supplementary file 2 [file hyp-77-1273-s002.pdf]

DATA SUPPLEMENT

Cardiovascular Effects of Unilateral Nephrectomy in Living Kidney Donors at Five Years

Short title: Cardiovascular effects of living kidney donation

Anna M. Price[1,2], William E. Moody[3], Victoria M. Stoll[1,3], Ravi Vijapurapu[1,3], Manvir K. Hayer[1,2], Luca Biasiolli[4], Chris J. Weston[5], Rachel Webster[6], Roman Wesolowski[7], Kirsty C. McGee[8], Boyang Liu[1,3], Shanat Baig[1,3], Luke C. Pickup[1,3], Ashwin Radhakrishnan[1,3], Jonathan P. Law[1,2], Nicola C. Edwards[9], Richard P. Steeds[1,3], Charles J. Ferro[1,2], Jonathan N. Townend[1,3]

Corresponding authors details:
Name: Dr Anna M. Price
Address: Cardiology Research Team, Room 19 Clinical Research Offices, Old Nuclear Medicine Department, Queen Elizabeth Hospital, Edgbaston, Birmingham, B15 2TH, United Kingdom
Phone: +44121 371 4624
Email: annaprice@doctors.org.uk

1. Institute of Cardiovascular Sciences, University of Birmingham, Birmingham, UK.
2. University Hospitals NHS Foundation Trust, Department of Nephrology, Birmingham, UK.
3. University Hospitals NHS Foundation Trust, Department of Cardiology, Birmingham, UK
4. Oxford Centre for Magnetic Resonance, University of Oxford, Oxford, UK
5. Institute of Immunology and Immunotherapy, University of Birmingham, Birmingham, UK.
6. University Hospitals NHS Foundation Trust, Department of Biochemistry, Birmingham, UK
7. University Hospitals NHS Foundation Trust, Department of Imaging, Birmingham, UK
8. Institute of Inflammation and Ageing, University of Birmingham, Birmingham, UK.
9. Green Lane Cardiovascular Service, Auckland, NZ

SUPPLEMENTAL TEXT

Supplemental methodology 1:

Cardiac magnetic resonance acquisition:

Studies were performed at 5 years using a 3T scanner (Magnetom, Skyra, Siemens, Germany). If contra-indications to 3T imaging were present a 1.5T (Magnetom Avanto, Siemens, Germany) machine was used.

Scan parameters for steady state free precession image (SSFP) cines were as follows: repetition time 45.48ms (TR); echo time 1.69 (TE); flip angle (FA); 65°; field of view (FOV) 340mm with a slice thickness of 7mm with 3mm gap over 25 phases per cardiac cycle.[1]

A breath-held SSFP motion corrected modified Look-Locker inversion recovery (MOLLI) sequence was used for native T1 mapping at the mid ventricular short axis level prior to the administration of 0.15mmol/kg of gadolinium contrast (Gadovist®, Bayer Healthcare Pharmaceuticals). A sampling scheme of 5(3)3 was used with a total breath hold of 11 R-R intervals. Parameters for MOLLI were: TR 280.56ms, TE 1.12ms, FA 35°, and FOV 360mm. For T2 mapping, 3 single shot images of the same mid ventricular short axis level were taken at the following T2 preparation times, 0ms, 30ms and 55ms. Standard T1- weighted gradient echo inversion recovery images of the vertical long axis, horizontal long axis and LV stack sequences were repeated for the assessment of late gadolinium enhancement 5-7 minutes after contrast administration. Post contrast MOLLI images were acquired using identical slice positions as native images using a 4(1)3(1)2 sampling scheme 15 minutes following administration of gadolinium.

Reproducibility assessment for left ventricular mass:
Intra-observer variability was assessed in 10 randomly selected studies, which were analysed twice by the same blinded observer (A.M.P.) within a 2 month period. Inter-study variability was assessed in ten subjects undergoing a repeat scan within one week of the original study. Inter-observer variability between the two independent reporters (A.M.P & W.E.M) was assessed in 20 subjects.

Supplemental methodology 2:

Methodology of aortic distensibility:

Aortic distensibility was assessed using software developed in Matlab version R2017a (Mathworks, USA).[2] The aortic lumen was detected and tracked throughout the cardiac cycle on cine magnetic resonance imaging (MRI).[2] The cross-sectional luminal area (mm^2) in systole (maximal area = A_{max}) and in diastole (minimal area = A_{min}) were estimated from an average of 3 measures. Aortic distensibility was calculated as:

$$\text{Aortic distensibility} = \frac{\left(\frac{\text{Amax -Amin}}{\text{Amin}}\right)}{\text{Pulse pressure}}$$

Pulse pressure was an average of 3 blood pressure readings taken immediately after the MRI sequence whilst the subject remained supine within the scanner.

Supplemental methodology 3:

Non-invasive measures of arterial stiffness and calculations:

Pulse wave analysis (PWA) and pulse wave velocity (PWV) were measured with the SphygmoCor device (Atcor Medical, Sydney, Australia). The augmentation index, corrected for a heart rate of 75 beats per minute (AI_{75}), was used for analysis.[3] PWV was adjusted for both mean arterial pressure (MAP) and heart rate (HR).[3] Path length was defined as the distance between the sternal notch and carotid pulse subtracted from the distance from the sternal notch to the femoral pulse.[3]

PWV was adjusted for mean arterial pressure and heart rate using a general linear regression model (PWV as dependent and MAP and HR as independent variables i.e. a multivariate model).[3] Calculations were as follows:

- Predicted PWV= Constant (from regression equation) + [(Unstandardized B coefficient (from regression model equation)* mean MAP of cohort)] + [(Unstandardized B coefficient (from regression model equation)*mean HR of cohort)].
- Adjusted PWV for an individual= Predicted PWV assuming mean MAP and HR + Unstandardized residual for the individual (from regression model).

Supplemental methodology 4:

This analysis was part of a post hoc analysis of blood pressure. Time was defined as the date of the baseline visit to the date of final follow up. A composite end-point defining a clinically significant increase in blood pressure was defined using 4 combined outcomes:

1. 24hr systolic blood pressure increase of 10 mmHg or more.
2. 24hr diastolic blood pressure increase of 5 mmHg or more.
3. 24hr ambulatory blood monitoring of 130/80 mmHg or more.
4. Receipt of anti-hypertensive medication.

An event was considered as the time to occurrence of the first of the listed outcomes above. Subjects that were receiving antihypertensive treatment at baseline were excluded from this analysis. At 5 years a total of 21 (47%) controls and 28 (56%) donors had reached the composite end-point. An interval censored cox regression was used for analysis using the icenReg package in R. The hazard ratio (HR) for hypertension using the combined outcome in donors relative to controls was increased but not significant, [HR 1.38 (95% confidence interval 0.74, 2.60), p=0.313].

SUPPLEMENTAL REFERENCES

1. Maceira AM, Prasad SK, Khan M, Pennell DJ. Normalized left ventricular systolic and diastolic function by steady state free precession cardiovascular magnetic resonance. *Journal of Cardiovascular Magnetic Resonance*. 2006;8:417-426
2. Biasiolli L, Hann E, Lukaschuk E, Carapella V, Paiva JM, Aung N, Rayner JJ, Werys K, Fung K, Puchta H, Sanghvi MM, Moon NO, Thomson RJ, Thomas KE, Robson MD, Grau V, Petersen SE, Neubauer S, Piechnik SK. Automated localization and quality control of the aorta in cine cmr can significantly accelerate processing of the uk biobank population data. *PLOS ONE*. 2019;14:e0212272
3. Townsend RR, Wilkinson IB, Schiffrin EL, Avolio AP, Chirinos JA, Cockcroft JR, Heffernan KS, Lakatta EG, McEniery CM, Mitchell GF, Najjar SS, Nichols WW, Urbina EM, Weber T. Recommendations for improving and standardizing vascular research on arterial stiffness. *Hypertension*. 2015;66:698-722
4. Kim PK, Hong YJ, Im DJ, Suh YJ, Park CH, Kim JY, Chang S, Lee H-J, Hur J, Kim YJ, Choi BW. Myocardial t1 and t2 mapping: Techniques and clinical applications. *Korean Journal of Radiology*. 2017;18:113-131
5. Mangion K, McDowell K, Mark PB, Rutherford E. Characterizing cardiac involvement in chronic kidney disease using cmr-a systematic review. *Current cardiovascular imaging reports*. 2018;11:2-2
6. Bull S, White S, Piechnik S, Flett A, Ferreira V, Loudon M, Francis J, Karamitsos T, Prendergast B, Robson M, Neubauer S, Moon J, Myerson S. Human non-contrast t1 values and correlation with histology in diffuse fibrosis. *Heart*. 2013;99:932 - 937
7. Cerqueira Manuel D, Weissman Neil J, Dilsizian V, Jacobs Alice K, Kaul S, Laskey Warren K, Pennell Dudley J, Rumberger John A, Ryan T, Verani Mario S. Standardized myocardial segmentation and nomenclature for tomographic imaging of the heart. *Circulation*. 2002;105:539-542
8. Edwards NC, Moody WE, Yuan M, Hayer MK, Ferro CJ, Townend JN, Steeds RP. Diffuse interstitial fibrosis and myocardial dysfunction in early chronic kidney disease. *American Journal of Cardiology*.2015;115:1311-1317

SUPPLEMENTAL TABLES

Table S1; Baseline demographics in those that attended follow up and were lost to follow up at the 5 year study visit.

**Variable**	Follow up at 5 years n=95	Lost to follow up n=29	p-value
**Donor**	50 (53)	18 (62)	0.249
**Age** (years)	46 ± 13	43 ± 11	0.230
**Male sex**	35 (37)	15 (51)	0.113
**Race**			
White	85 (90)	2 (83)	0.288
Asian	7 (7)	3 (10)	
Black	3 (3)	2 (7)	
**Cardiovascular risk factors**			
Hypercholesterolemia	5 (5)	1 (3)	0.557
Diabetes	0 (0)	0 (0)	-
Hypertension	5 (5)	2 (7)	0.534
Stroke/TIA	0 (0)	0 (0)	-
IHD	0 (0)	0 (0)	-
**Family history**			
Cardiovascular	27 (28)	3 (10)	**0.048**
**Smoking history**			
Current smoker	6 (6)	5 (17)	0.126
Ex-smoker	27 (28)	7 (24)	0.466
**Antihypertensive usage**			
ACEi	3 (3)	1 (3)	0.671
β blocker	1 (1)	1 (3)	0.423
Calcium channel	2 (2)	0 (0)	0.564
**Other medication usage**			
Statin	5 (5)	1 (3)	0.557
Levothyroxine	3 (3)	2 (7)	0.333
Aspirin	0 (0)	0 (0)	-
NSAIDs	2 (2)	0 (0)	0.585

Data are displayed as mean ± SD or number of patients (percentage). Analysis was carried out using independent samples *t* tests for continuous data and fishers exact tests or chi squared tests for categorical data.

ACEi; Angiotensin converting enzyme inhibitor, BMI; Body mass index. IHD; Ischaemic heart disease. NSAIDS; Non-steroidal anti-inflammatories. SD; Standard deviation. TIA; Transient ischaemic event

Table S2; Incidental findings during the study.

**Healthy controls**	n=1 Hypertension requiring medication.<br>n=1 Large pericardial cyst.<br>n=1 Prolapsing mitral valve under surveillance.<br>n=1 Abnormal liver function.<br>n=1 Iron deficiency anaemia.<br>n=1 Dilated aorta.<br>n=1 Asymptomatic 1st degree heart block.
**Living kidney donors**	n=1 Borderline diabetes.<br>n=2 Breast cysts requiring referral.<br>n=1 Iron deficiency anaemia.<br>n=1 Asymptomatic 1st degree heart block.<br>n=1 1st degree block and syncope requiring loop recorder.<br>n=1 Atrial fibrillation.

Table S3; Haematological and biochemical effects.

Variable	Baseline	Within-group change * (baseline to 12 months)	Within-group change * (baseline to 5 years)	Between-group difference † (for 5 year change)
Hemoglobin (g/dl)				
Donor	13.7 ± 1.2	-0.35 (-0.57, -0.13)	+0.00 (-0.28, 0.29)	-0.07 (-0.44, 0.31)
Control	13.5 ± 1.2	+0.01 (-0.21, 0.24)	+0.07 (-0.16, 0.30)	
Creatinine (µmol/L)				
Donor	73 ± 14	+27.97 (24.46, 31.49)	+23.14 (19.40, 26.87)	+22.28 (17.65, 26.90)
Control	71 ± 13	+1.17 (-1.48, 3.84)	+0.86 (-1.70, 3.42)	
eGFR (ml/min/1.73m^2)				
Donor	95 ± 15	-29.20 (-32.09, -26.32)	-27.64 (-31.09, -24.18)	-22.74 (-27.39, -18.07)
Control	99 ± 16	-2.12 (-5.63, 1.37)	-4.90 (-8.02, -1.78)	
ACR (≥3mg/mmol) ‖				
Donor	1 (2.0)	3 (6.4)	6 (12.2)	5.26 (0.66, 42.02)
Control	1 (2.2)	0 (0.0)	1 (2.3)	
Corrected calcium (mmol/L)				
Donor	2.20 ± 0.98	+0.01 (-0.01, 0.05)	+0.08 (0.05, 0.11)	+0.03 (-0.00, 0.06)
Control	2.19 ± 0.08	+0.02 (-0.01, 0.05)	+0.05 (0.03, 0.08)	
Phosphate (mmol/L)				
Donor	1.07 ± 0.16	-0.02 (-0.06, 0.01)	-0.05 (-0.10, -0.00)	-0.05 (-0.14, 0.02)
Control	1.09 ± 0.16	+0.04 (-0.06, 0.15)	+0.00 (-0.06, 0.08)	

Parathyroid hormone (pmol/L)				
Donor	4.4 ± 1.36	+1.31 (0.72, 1.89)	+0.78 (0.26, 1.31)	+0.33 (-0.37, 1.03)
Control	4.28 ± 1.19	+0.29 (-0.10, 0.69)	+0.45 (-0.03, 0.95)	
Vitamin D (nmol/L)				
Donor	50 ± 27	+7.12 (-0.36. 14.61)	+5.20 (-4.55, 14.95)	-2.17 (-16.66, 12.31)
Control	53 ± 22	+0.62 (-6.90, 8.15)	+7.37 (-3.60, 18.35)	
FGF23 (RU/ml) §				
Donor	72.44 [63.09, 83.17]	×1.23 (1.09, 1.41)	×1.02 (0.91, 1.17)	×1.25 (1.02, 1.54)
Control	75.85 [63.09, 89.12]	×1.04 (0.89, 1.28)	×0.81 (0.69, 0.97)	
Urate (µmol/L)				
Donor	267 ± 74	+55.52 (44.44, 66.59)	+68.08 (53.64, 82.53)	+77.59 (55.25, 99.94)
Control	288 ± 54	-1.13 (-11.90, 9.62)	-9.51 (-27.31, 8.29)	
hsCRP (mg/L) §				
Donor	0.87 [0.60, 1.23]	×1.65 (1.20, 2.23)	×2.18 (1.58, 3.01)	×1.38 (0.93, 2.04)
Control	0.83 [0.61, 1.12]	×1.14 (0.91, 1.44)	×1.58 (1.24, 2.04)	
Total Cholesterol (mmol/L)				
Donor	5.4 ± 1.1	-0.01 (-0.27, 0.23)	-0.17 (-0.38. 0.02)	-0.10 (-0.42, 0.21)
Control	5.0 ± 1.0	-0.00 (-0.21, 0.20)	-0.07 (-0.32, 0.17)	
LDL cholesterol (mmol/L)				
Donor	3.1 ± 1.0	+0.07 (-0.10, 0.24)	-0.13 (-0.34, 0.07)	+0.11 (-0.23, 0.45)
Control	2.9 ± 0.9	-0.09 (-0.30, 0.11)	-0.24 (-0.53, 0.03)	

HDL cholesterol (mmol/L)				
Donor	1.7 ± 0.5	-0.03 (-0.10, 0.04)	+0.00 (-0.15, 0.17)	+0.00 (-0.18, 0.20)
Control	1.6 ± 0.4	+0.02 (-0.03, 0.09)	+0.00 (-0.10, 0.11)	
Triglycerides (mmol/L) §				
Donor	1.11 [0.97, 1.28]	×1.07 (0.97, 1.20)	×1.14 (1.00, 1.34)	×0.97 (0.79, 1.20)
Control	0.96 [0.85, 1.09]	×1.02 (0.93, 1.17)	×1.17 (1.00, 1.38)	
hsTNT ≥ 5(ng/L) ‖				
Donor	3 (7.0)	10 (25.0)	28 (59.6)	1.25 (0.84, 1.85)
Control	4 (10.0)	1 (3.1)	20 (48.8)	
hsTNT ≥ 14(ng/L) ‖				
Donor	0 (0)	0 (0)	2 (4.7)	4.69 (0.23, 95.2)
Control	0 (0)	0 (0)	0 (0)	
NT pro BNP (pmol/L) §				
Donor	1.17 [0.61, 2.18]	×1.69 (0.79, 3.71)	×0.60 (0.30, 1.14)	×1.54 (0.61, 3.98)
Control	1.21 [0.58, 2.51]	×0.72 (0.42, 1.23)	×0.38 (0.19, 0.75)	

Data are displayed as mean ± SD or geometric mean [95% CI] at baseline for the whole cohort. Mean (95% CI) are displayed for within-group change and between-group difference.

*Within-group change and 95% CI are from paired analyses.

† Between-group difference and 95% CI are from unpaired analyses.

§ Non-parametric data was log10 transformed prior to analysis. Values for within-group change and between group differences are displayed as antilogged values with (95% CI). These values are multipliers.

|| For categorical data the baseline prevalence is presented and the within group change is the incidence at 12 months and 5 years in those who did not have the condition at baseline. Prevalence and incidence are given as counts (percentage). The between-group difference is the relative risk (incidence for donors relative to controls) and 95% confidence intervals.

ACR; Albumin creatinine ratio. CI; Confidence interval. CRP; C - reactive protein. eGFR; estimated glomerular filtration rate. FGF23; fibroblast growth factor 23. HDL; high-density lipoprotein. LDL; low-density lipoprotein. NT pro BNP; n-terminal pro brain natriuretic peptide. hsTNT; high sensitivity troponin. SD: Standard deviation.

Table S4; Multiple linear regression model of variables influencing change in absolute left ventricular mass (g) at 5 years in the whole cohort.

	Multivariable analysis			Final model analysis		
	**β**	**CI**	***p-value***	**B**	**CI**	***p-value***
Donor	*	*	*	0.668 †	-6.675, 5.339 †	0.824
Follow up (mths.)	*	*	*	0.217	-0.147, 0.580	0.237
Age at baseline (yrs.)	*	*	*	0.212 ‡	-0.225, 0.648 ‡	0.335
Interaction between age and donor/control status	*	*	*	-0.484	-0.225, 0.648	0.065
Male sex	2.458	-2.675, 7.591	0.343			
Baseline LVM (g)	-0.018	-0.110, 0.073	0.689			
Change in eGFR (ml/min/1.73m^2)	-0.029	-0.265, 0.208	0.811			
Change in BMI (kg/m^2)	0.897	-0.050, 1.844	0.063			
Change in office MAP (mmHg)	0.148	-0.156, 0.451	0.336			
Change in day SBP (mmHg)	0.459	0.063, 0.855	**0.024**	0.459	0.063, 0.855	**0.024**
Change in day DBP (mmHg)	0.312	-0.197, 0.821	0.224			
Change in adjusted PWV (m/s)	1.057	-1.493, 3.608	0.411			
Change in uric acid (µmol/L)	0.037	-0.014, 0.087	0.154			
Change in PTH (pmol/L)	-0.502	-2.174, 1.709	0.649			

Change in absolute left ventricular mass (g) was the dependent variable in all analyses. A general linear model was used to test for the interaction between each variable and donor/control status. The only significant interaction was between age at baseline and donor/control status and this was therefore incorporated into all models. For each explanatory variable the values reported under multivariable analysis are from a linear regression model that also included follow up time, donor/control status, age at baseline and the interaction between age and donor/control

status. As the only significant variable in the multivariable analyses, change in day SBP was chosen for the final model, which also included follow up time, donor/control status, age at baseline and the interaction between age and donor/control status. None of the other variables were significant when added to this model.

* Indicates variables included in all multivariable analysis models.

†The estimated difference in means (donor minus control) is given for an individual of mean age (46 yrs.).
‡ Value for a control.

BMI; Body mass index. β; Unstandardized beta coefficient. CI; Confidence interval. eGFR; estimated glomerular filtration rate. DBP; Diastolic blood pressure. LVM; Left ventricular mass. MAP; mean arterial pressure. Mths: Months. PTH; Parathyroid hormone. PWV; Pulse wave velocity adjusted for MAP and HR. SBP: Systolic blood pressure. Yrs: Years. Coefficients are given per unit change e.g. per year for age.

Table S5; Reproducibility for primary end-point

Reproducibility was assessed by calculating mean bias and 95% limits of agreement. p-values are derived from paired t tests comparing each rater.

		**Mean bias**	**p**	**95% limits of agreement**		**ICC**
Absolute LVM (g)	Inter-study	-1.11 ± 2.3	0.153	-5.52	3.32	0.99 (0.98, 0.99)
	Intra-observer	-0.21 ± 3.1	0.834	-6.34	5.92	0.99 (0.96, 0.99)
	Inter-observer	-1.15 ± 2.6	0.066	-6.24	3.94	0.99 (0.97, 0.99)

SUPPLEMENTAL FIGURES

Figure S1; Methodology of T1 and T2 mapping.

Native T1 times reflect changes in myocardial tissue; high values can occur due to increased water content or increases in interstitial space (e.g. fibrosis).[4] T1 time has been demonstrated to be elevated in both end stage renal disease and in early stage chronic kidney disease compared to controls.[5] In other cardiac conditions such as aortic stenosis T1 has been quantitatively associated with biopsy proven myocardial fibrosis.[6] Extracellular volume (ECV) is determined using both native and post contrast T1 mapping and varies less across scanner field strengths.[4] ECV has also been associated with biopsy proven fibrosis with greater sensitivity.[4] T2 mapping is a non-contrasted CMR technique measuring transverse relaxation time and is an index of myocardial water content.[4]

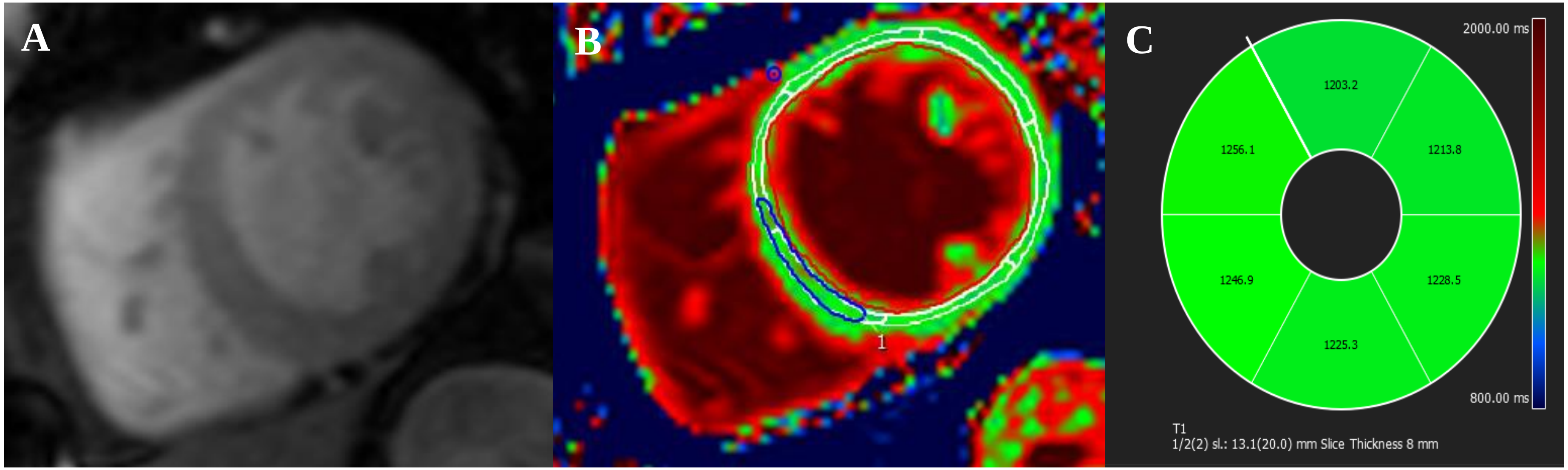

a. **Demonstration of the raw uncorrected MOLLI image:** The uncorrected raw images of the mid ventricular slices were reviewed initially for artefact across the myocardium and any motion. If the raw images were suitable, epicardial and endocardial contours were then defined on the motion-corrected raw images, taking care to exclude obvious blood pool.
b. **A native T1 map of the same LV slice:** Contours were then transferred to the generated T1 map. The T1 map is colour coded according to time: 800ms dark blue, 1000ms light blue, 1250ms green, 1400ms red and 2000ms dark red. The contours can be seen on this image. Using an inferior and superior insertion point for reference the myocardium was segmented, giving six segments for each slice (seen here

in white).[7] A 20% offset setting was used. Segments previously identified on the raw images with artefact, late gadolinium enhancement or blood pools were excluded from final analysis.

c. **Segmental T1 times:** T1 times for each segment are generated as American Heart Association (AHA) segments.[7] Blood time was also derived from a circular contour within the blood pool taking care to avoid papillary muscles or trabeculation. For those who received gadolinium the process was repeated using post contrast images of the corresponding slice. ECV was calculated as previously described.[8] The haematocrit used for the ECV calculation was taken from venous blood on the day of the CMR. Times are given as global values: average of all segments suitable for analysis without artefact. T2 times were recorded using the same methodology - a 20% offset and segmenting each slice into AHA segments.
